# Sociodemographic inequality in COVID-19 vaccination coverage among elderly adults in England: a national linked data study

**DOI:** 10.1136/bmjopen-2021-053402

**Published:** 2021-07-23

**Authors:** Vahe Nafilyan, Ted Dolby, Cameron Razieh, Charlotte Hannah Gaughan, Jasper Morgan, Daniel Ayoubkhani, Sarah Walker, Kamlesh Khunti, Myer Glickman, Thomas Yates

**Affiliations:** 1 Health Analysis and Life Event, Office for National Statistics, Newport, UK; 2 Faculty of Public Health, Environment and Society, London School of Hygiene & Tropical Medicine, London, UK; 3 Diabetes Research Centre, University of Leicester, Leicester, UK; 4 NIHR Leicester Biomedical Research Centre, University of Leicester, Leceister, UK; 5 University Hospitals of Leicester NHS Trust, Leicester, UK; 6 Methodology Division, Office for National Statistics, Newport, UK; 7 Nuffield Department of Medicine, University of Oxford, Oxford, UK

**Keywords:** COVID-19, infection control, epidemiology

## Abstract

**Objective:**

To examine inequalities in COVID-19 vaccination rates among elderly adults in England.

**Design:**

Cohort study.

**Setting:**

People living in private households and communal establishments in England.

**Participants:**

6 655 672 adults aged ≥70 years (mean 78.8 years, 55.2% women) who were alive on 15 March 2021.

**Main outcome measures:**

Having received the first dose of a vaccine against COVID-19 by 15 March 2021. We calculated vaccination rates and estimated unadjusted and adjusted ORs using logistic regression models.

**Results:**

By 15 March 2021, 93.2% of people living in England aged 70 years and over had received at least one dose of a COVID-19 vaccine. While vaccination rates differed across all factors considered apart from sex, the greatest disparities were seen between ethnic and religious groups. The lowest rates were in people of black African and black Caribbean ethnic backgrounds, where only 67.2% and 73.8% had received a vaccine, with adjusted odds of not being vaccinated at 5.01 (95% CI 4.86 to 5.16) and 4.85 (4.75 to 4.96) times greater than the white British group. The proportion of individuals self-identifying as Muslim and Buddhist who had received a vaccine was 79.1% and 84.1%, respectively. Older age, greater area deprivation, less advantaged socioeconomic position (proxied by living in a rented home), being disabled and living either alone or in a multigenerational household were also associated with higher odds of not having received the vaccine.

**Conclusion:**

Research is now urgently needed to understand why disparities exist in these groups and how they can best be addressed through public health policy and community engagement.

**Strengths and limitations of this study**

- The main strength of our population-level dataset is the availability of a wide range of sociodemographic characteristics not included in electronic health records, allowing for a detailed examination of inequalities in vaccination coverage.
- We presented vaccination rates and Odds Ratios for non-vaccination adjusted for a range of factors to understand further inequalities in vaccination coverage.
- The main limitation is that most demographic and socioeconomic characteristics were derived from the 2011 Census and therefore are 10 years old.
- Because the dataset is based on the 2011 Census, it excluded people living in England in 2011 but not taking part in the 2011 Census, respondents who could not be linked to the 2011–2013 National Health Service Patient Register and recent migrants.

## Introduction

The UK began an ambitious vaccination programme to combat the COVID-19 pandemic on 8 December 2020; by 24 April 2021, 64% of the UK adult population have received their first of the dose.^1^

Previous research demonstrates that vaccination rates tend to be lower among certain ethnic groups, and in areas of higher deprivation.^2–4^ Existing evidence suggests that COVID-19 vaccination rates differ by level of area deprivation, certain underlying health conditions and ethnicity.^5^ Far less is known about how COVID-19 vaccination uptake varies by sociodemographic factors, such as religious affiliation, individual socioeconomic status, living in multigenerational household or disability status, factors disproportionately associated with SARS-CoV-2 infection. Understanding which sociodemographic, economic and cultural factors are associated with low vaccination rates has major implications for designing policies that help maximise the vaccination campaign coverage.

This study investigates inequality in vaccination rates among adults aged ≥70 years in England, using population-level administrative records linked to the 2011 Census. This enables examination of a wide range of sociodemographic characteristics, currently lacking in previously published studies, in particular ethnicity, religion, different measures of socioeconomic position and those who report being disabled.

## Methods

### Study data

We linked vaccination data from the National Immunisation Management System (NIMS) to the Office for National Statistics (ONS) Public Health Data Asset (PHDA) based on National Health Service (NHS) number. The ONS PHDA is a linked dataset combining the 2011 Census, mortality records, the General Practice Extraction Service data for pandemic planning and research and the Hospital Episode Statistics. To obtain NHS numbers for the 2011 Census, we linked the 2011 Census to the 2011–2013 NHS Patient Registers using deterministic and probabilistic matching, with an overall linkage rate of 94.6%. All subsequent linkages were performed based on NHS numbers.

The study population consisted of people aged ≥70 years, alive on 15 March 2020, who were residents in England, registered with a general practitioner and enumerated at the 2011 Census. Of 6 605 315 adults aged ≥70 years who received a first dose of a COVID-19 vaccine in NIMS, 6 242 384 (94.5%) were linked to the ONS PHDA.

### Outcome

The main outcome was having received at least a first dose of a COVID-19 vaccine by 15 March 2021, as recorded in the NIMS data available on 31 March 2021. Phase 1 of the vaccination policy for England aimed to offer a first vaccination appointment to all those ≥70 years by 15 February, and we allowed a further month to ensure full coverage.

### Exposures and covariates

This dataset combines comprehensive sociodemographic information from the 2011 Census with a detailed medical history from clinical records. All individual-level sociodemographic characteristics (ethnic group, religious affiliation, disability status, educational attainment) came from the 2011 Census. We used a 10-category ethnic group classification (White British, Bangladeshi, Black African, Black Caribbean, Chinese, Indian, Mixed, Other, Pakistani, White other). Self-reported religious group, place of residence (region within England, private or care home) and area-based deprivation (Index of Multiple Deprivation^6^) were derived based on the 2019 Patient Register. Comorbidities were defined as in the QCovid risk prediction model, a model used to assess the risk of severe COVID-19 outcomes in the general population, used to inform the prioritisation of the vaccination campaign.^7^ All variables included in this analysis are listed in table 1.

**Table 1:** Variables used in the analyses

Variable	Coding	Source
Vaccinated	Received a first dose of a COVID-19 vaccine by 15 March 2020	NIMS
Age	Third-order polynomial	2011 Census
Sex	Female, male	2011 Census
Ethnicity	White British, Bangladeshi, Black African, Black Caribbean, Chinese, Indian, Mixed, Other, Pakistani, White other	2011 Census
Religious affiliation	Christian, Buddhist, Hindu, Jewish, Muslim, no religion, other religion, religion not stated, Sikh	2011 Census
Region	Dummy variables representing region of residence	2019 NHS Patient Register
Rural–urban classification	Urban, rural	2019 NHS Patient Register
Index of Multiple Deprivation	Dummy variables representing quintiles of deprivation	2019 NHS Patient Register
Household tenure	Own, social rented, private rented, other	2011 Census
Level of highest qualification	Degree, A-level or equivalent, GCSE or equivalent, no qualification, other	2011 Census
Disability	Non-disabled, disabled (limited a little), disabled (limited a lot)	2011 Census
Body mass index (kg/m^2^)	<18.5, 18.5–25, 25–30, ≥30, missing	GPES
Chronic kidney disease (CKD)	No CKD, CKD3, CKD4, CKD5	GPES
Learning disability	No learning disability, Down’s syndrome, other learning disability	GPES
		
Cancer and immunosuppression	Dummies for blood cancer, solid organ transplant, prescribed immunosuppressant medication by GP, prescribed leukotriene or long-acting beta blockers, prescribed regular prednisolone	GPES
Other conditions	Diabetes, chronic obstructive pulmonary disease, asthma, rare pulmonary diseases, pulmonary hypertension or pulmonary fibrosis, coronary heart disease, stroke, atrial fibrillation, congestive cardiac failure, venous thromboembolism, peripheral vascular disease, congenital heart disease, dementia, Parkinson’s disease, epilepsy, rare neurological conditions, cerebral palsy, severe mental illness (bipolar disorder, schizophrenia, severe depression), osteoporotic fracture, rheumatoid arthritis or systemic lupus erythematosus, cirrhosis of the liver	GPES/HES

GCSE, General Certificate of Secondary Education; GP, general practitioner; GPES, General Practice Extraction Service; HES, Hospital Episode Statistics; NHS, National Health Service; NIMS, National Immunisation Management System.

### Statistical analyses

First, we estimated the first dose vaccination rates by a range of demographic and socioeconomic characteristics. Second, to understand the drivers of the observed differences in vaccination rates, we used logistic regression to estimate the odds of not having received a first dose of a COVID-19 vaccine. For each exposure, we compared ORs from models adjusted for different sets of covariates. We estimated unadjusted ORs, ORs adjusted for sex and age, and ORs adjusted for all geographical and sociodemographic characteristics, disability status and pre-existing conditions. All analyses were conducted using R V.3.5

### Patient and public involvement

No patient involved.

## Results

Our study population included 6 655 672 adults aged ≥70 years who lived in England. A total of 55.2% were women and the mean age was 78.8 (SD: 6.5) years; 91.6% identified themselves as White British, 78.5% as Christian. A total of 82.5% owned their home (table 2). By 15 March 2021, 93.2% of people living in England aged 70 years and over had received at least one dose of a COVID-19 vaccine.

**Table 2:** Characteristics of the study population

Variable	Level	Count (%)
Vaccinated		6 202 780 (93.2)
Sex	Female	3 672 314 (55.2)
	Male	2 983 358 (44.8)
Age	Mean (SD)	78.8 (6.5)
Ethnicity	Bangladeshi	11 522 (0.2)
	Black African	21 535 (0.3)
	Black Caribbean	52 883 (0.78)
	Chinese	18 452 (0.38)
	Indian	103 564 (1.6)
	Mixed	24 637 (0.34)
	Other	65 241 (1.0)
	Pakistani	39 723 (0.6)
	White British	6 095 276 (91.6)
	White other	222 839 (3.4)
Religion	Buddhist	16 403 (0.3)
	Christian	5 221 392 (78.5)
	Hindu	61 634 (0.9)
	Jewish	39 800 (0.6)
	Muslim	86 841 (1.3)
	No religion	725 695 (10.9)
	Other religion	22 327 (0.3)
	Religion not stated	449 781 (6.8)
	Sikh	31 799 (0.5)
IMD quintile	1 (most deprived)	913 809 (13.7)
	2	1 140 651 (17.1)
	3	1 407 155 (21.1)
	4	1 560 023 (23.4)
	5 (least deprived)	1 634 034 (24.6)
Household tenure	Owned	5 488 126 (82.5)
	Private rented	273 707 (4.1)
	Social rented	778 867 (11.7)
	Other (eg, live rent free)	114 972 (1.7)
Rural–urban	Rural	6 005 144 (82.4)
	Urban	304 412 (4.2)
Household composition	2 elderly	847 508 (11.6)
	1 elderly	128 083 (1.8)
	Care home	436 211 (6.0.)
	Missing household	9035 (0.1)
	Multigenerational	620 167 (8.5)
	Other (3+ adults)	714 211 (9.8)

Adults aged 70 years or over, living in England, alive on 15 March 2021.

IMD, Index of Multiple Deprivation.

Table 3 shows vaccination rates by demographic and socioeconomic factors, as well as ORs from different models. Vaccination rates differed across all factors considered, apart from sex. The lowest rates were in people of Black African and Black Caribbean ethnic backgrounds where only 67.2% and 73.9% had received a vaccine. Adjusting for differences in geography, sociodemographic factors and underlying health conditions did not fully explain the lower probability of having received the vaccine among ethnic minority groups. Compared with people of white British ethnicity, the fully adjusted OR for Black African individuals was 5.01 (95% CI 4.86 to 5.16), while the unadjusted OR was 7.62 (7.40 to 7.84), suggesting that geography, sociodemographic factors and pre-pandemic health only explain about 40% of the elevated odds of not being vaccinated.

**Table 3:** Vaccination rates and ORs for not being vaccinated by sociodemographic characteristics

Exposure	Group	Vaccination rate	OR (model 1)	OR (model 2)	OR (model 3)
Age group	70–74	90.9 (90.8 to 90.9)	1 (ref)	1 (ref)	1 (ref)
	75–79	93.8 (93.8 to 93.9)	0.65 (0.65 to 0.66)	0.65 (0.65 to 0.66)	0.66 (0.65 to 0.66)
	80–84	95.6 (95.5 to 95.6)	0.46 (0.46 to 0.46)	0.46 (0.46 to 0.46)	0.45 (0.45 to 0.46)
	85–89	95.1 (95.0 to 95.1)	0.51 (0.51 to 0.52)	0.51 (0.51 to 0.52)	0.51 (0.51 to 0.52)
	90–94	94.0 (93.9 to 94.1)	0.63 (0.62 to 0.64)	0.63 (0.62 to 0.64)	0.64 (0.63 to 0.65)
	95–99	92.3 (92.1 to 92.5)	0.83 (0.81 to 0.85)	0.82 (0.80 to 0.85)	0.82 (0.80 to 0.84)
	100+	85.5 (84.8 to 86.1)	1.69 (1.61 to 1.78)	1.68 (1.60 to 1.77)	1.60 (1.52 to 1.68)
Sex	Female	93.2 (93.2 to 93.2)	1 (ref)	1 (ref)	1 (ref)
	Male	93.2 (93.2 to 93.2)	1.00 (1.00 to 1.01)	0.98 (0.98 to 0.99)	1.03 (1.02 to 1.03)
Disability	Not limited	93.5 (93.5 to 93.5)	1 (ref)	1 (ref)	1 (ref)
	Limited a little	93.4 (93.3 to 93.4)	1.02 (1.01 to 1.03)	1.12 (1.11 to 1.13)	1.08 (1.07 to 1.08)
	Limited a lot	91.3 (91.2 to 91.3)	1.37 (1.36 to 1.38)	1.47 (1.46 to 1.48)	1.29 (1.28 to 1.30)
Ethnicity	White British	94.0 (94.0 to 94.0)	1 (ref)	1 (ref)	1 (ref)
	Bangladeshi	82.7 (82.0 to 83.4)	3.26 (3.11 to 3.42)	3.48 (3.31 to 3.65)	2.56 (2.43 to 2.69)
	Black African	67.2 (66.6 to 67.8)	7.62 (7.40 to 7.84)	7.59 (7.37 to 7.81)	5.01 (4.86 to 5.16)
	Black Caribbean	73.8 (73.4 to 74.2)	5.55 (5.44 to 5.66)	6.35 (6.22 to 6.47)	4.85 (4.75 to 4.96)
	Chinese	82.8 (82.3 to 83.4)	3.23 (3.11 to 3.36)	3.11 (2.99 to 3.23)	2.64 (2.54 to 2.75)
	Indian	90.9 (90.7 to 91.0)	1.57 (1.54 to 1.60)	1.55 (1.52 to 1.59)	1.35 (1.32 to 1.38)
	Mixed	85.3 (84.9 to 85.7)	2.69 (2.60 to 2.79)	2.67 (2.58 to 2.77)	2.21 (2.14 to 2.30)
	Other	82.9 (82.6 to 83.2)	3.22 (3.15 to 3.29)	3.12 (3.05 to 3.18)	2.44 (2.39 to 2.50)
	Pakistani	79.6 (79.2 to 80.0)	3.99 (3.89 to 4.09)	4.12 (4.02 to 4.22)	3.59 (3.50 to 3.68)
	White other	87.7 (87.5 to 87.8)	2.20 (2.17 to 2.23)	2.24 (2.21 to 2.27)	1.93 (1.90 to 1.95)
IMD quintile	1 (most deprived)	90.6 (90.6 to 90.7)	1.77 (1.76 to 1.79)	1.76 (1.75 to 1.78)	1.60 (1.59 to 1.62)
	2	92.1 (92.0 to 92.1)	1.48 (1.46 to 1.49)	1.47 (1.46 to 1.48)	1.34 (1.33 to 1.35)
	3	93.4 (93.4 to 93.5)	1.20 (1.19 to 1.22)	1.20 (1.19 to 1.21)	1.17 (1.16 to 1.18)
	4	94.0 (94.0 to 94.0)	1.09 (1.08 to 1.10)	1.09 (1.08 to 1.10)	1.09 (1.08 to 1.10)
	5 (least deprived)	94.5 (94.4 to 94.5)	1 (ref)	1 (ref)	1 (ref)
Religion	Christian	93.8 (93.8 to 93.9)	1 (ref)	1 (ref)	1 (ref)
	Buddhist	84.1 (83.5 to 84.7)	2.88 (2.76 to 3.01)	2.63 (2.52 to 2.74)	2.03 (1.95 to 2.12)
	Hindu	91.5 (91.2 to 91.7)	1.43 (1.39 to 1.47)	1.39 (1.35 to 1.43)	1.03 (1.00 to 1.06)
	Jewish	93.1 (92.8 to 93.3)	1.13 (1.09 to 1.18)	1.12 (1.08 to 1.17)	0.94 (0.90 to 0.97)
	Muslim	79.1 (78.9 to 79.4)	4.02 (3.95 to 4.09)	4.04 (3.97 to 4.11)	2.74 (2.69 to 2.79)
	No religion	91.9 (91.9 to 92.0)	1.34 (1.33 to 1.35)	1.25 (1.23 to 1.26)	1.23 (1.22 to 1.24)
	Other religion	85.4 (84.9 to 85.8)	2.61 (2.51 to 2.71)	2.41 (2.32 to 2.50)	2.15 (2.07 to 2.23)
	Religion not stated	91.5 (91.4 to 91.6)	1.42 (1.41 to 1.44)	1.40 (1.38 to 1.41)	1.35 (1.33 to 1.36)
	Sikh	91.6 (91.3 to 91.9)	1.39 (1.34 to 1.45)	1.35 (1.30 to 1.41)	1.07 (1.03 to 1.11)
Household tenure	Owned	94.0 (93.9 to 94.0)	1 (ref)	1 (ref)	1 (ref)
	Other	91.1 (90.9 to 91.2)	1.52 (1.49 to 1.55)	1.55 (1.52 to 1.58)	1.49 (1.46 to 1.52)
	Private rented	88.4 (88.2 to 88.5)	2.05 (2.02 to 2.07)	1.96 (1.94 to 1.99)	1.81 (1.79 to 1.83)
	Social rented	89.9 (89.8 to 89.9)	1.75 (1.73 to 1.76)	1.77 (1.76 to 1.79)	1.60 (1.59 to 1.61)
Rural–urban	Rural	94.5 (94.4 to 94.5)	1 (ref)	1 (ref)	1 (ref)
	Urban	92.8 (92.8 to 92.8)	1.33 (1.32 to 1.34)	1.34 (1.33 to 1.35)	1.12 (1.12 to 1.13)
Household composition	2 elderly	94.5 (94.4 to 94.5)	1 (ref)	1 (ref)	1 (ref)
	1 elderly	92.5 (92.5 to 92.6)	1.38 (1.37 to 1.39)	1.53 (1.52 to 1.54)	1.32 (1.31 to 1.33)
	Care home	94.9 (94.8 to 95.0)	0.92 (0.89 to 0.95)	1.10 (1.06 to 1.13)	0.89 (0.86 to 0.91)
	Multigenerational	90.3 (90.3 to 90.4)	1.83 (1.82 to 1.85)	1.77 (1.76 to 1.79)	1.39 (1.38 to 1.40)
	Other (3+ adults)	90.0 (89.6 to 90.3)	1.91 (1.84 to 1.97)	1.81 (1.75 to 1.88)	1.54 (1.49 to 1.60)

Adults aged 70 years or over, living in England, alive on 15 March 2021. Model 1: unadjusted; model 2: adjusted for sex and age (cubic splines); model 3: adjusted for sex, age (cubic splines), care home status, rural/urban, region, ethnicity (except when looking at religion as an exposure), IMD quintile (except when looking at household tenure as an exposure), disability, BMI and comorbidities. See table 1 for more details on the variables included in the models.

BMI, body mass index; IMD, Index of Multiple Deprivation.

Vaccination rates also varied markedly across religious groups. While 93.8% of Christians had been vaccinated, only 79.1% of Muslims and 84.1% of Buddhists had been vaccinated. Stark differences remained after adjustment for other factors, with an adjusted OR of not being vaccinated of 2.74 (2.69 to 2.79) for Muslims and 2.03 (1.95 to 2.12) for Buddhists, compared with Christians.

Greater area deprivation, less advantaged socioeconomic position (proxied by living in a rented home), being disabled and living either alone or in a multigenerational household were also associated with low vaccination rates, even when adjusting for other factors (table 3). These differences were less pronounced than the differences between ethnic groups or religious affiliations.

## Discussion

### Main findings

Our analysis using whole population-level linked data in England suggests that first dose vaccination rates in adults aged ≥70 years differed markedly by ethnic group and self-reported religious affiliation. The percentage of people vaccinated was lower among all minority ethnic groups compared with the white British population, with the lowest vaccination rates observed among Black African, Black Caribbean, Bangladeshi and Pakistani individuals. In addition, lower vaccination rates were reported among individuals who identified as Muslim and Buddhist. While some differences were found by deprivation, household factors, disability status and other sociodemographic factors, these were less pronounced compared with ethnicity or religious affiliation.

### Comparison with other studies

Few studies have investigated how COVID-19 vaccination coverage varies by a wide range of sociodemographic characteristics. Our results on ethnicity and area deprivation are consistent with one previous study based on clinical records for 40% of patients in England.^3^ In addition, our results confirm studies showing that influenza, shingles and pneumococcal vaccination are patterned by similar factors, including ethnicity, deprivation and household size.^8^ Pre-pandemic, religion and culture have been postulated to be important factors in determining vaccination uptake^9^; our results extend this by showing that self-reported religious affiliation is an important factor in COVID-19 vaccine uptake. Differences in vaccination rate and potential vaccination hesitancy between religious groups may not be based on religious beliefs, but rather reflect safety and other concerns,^10^ or, given high infection rates in some of these groups,^11^ beliefs that vaccination is not needed after natural infection. We also find that vaccination rates vary by individual characteristics not reported in previous studies, such as household tenure (a proxy for socioeconomic status), household composition and disability status.

### Strengths and limitation

The primary study strength is using nationwide linked population-level data from clinical records and the 2011 Census. Unlike studies based solely on electronic health records, we examined a wide range of sociodemographic characteristics. Unlike surveys, we can precisely estimate vaccination rates and ORs for small groups. The main limitation is that most demographic and socioeconomic characteristics are derived from the 2011 Census and therefore are 10 years old. However, we focus primarily on characteristics that are unlikely to change over time, such as ethnicity or religion, or likely to be stable for our population (adults aged ≥70 years), such as household tenure. However, for the characteristics likely to change over time, such as disability status, the time difference may introduce some bias into the estimates, although this would be expected to dilute differences, since we are most likely missing some long-term health conditions. Care home residency and area deprivation were derived from the 2019 Patient Register and are therefore not subject to the same biases. Another limitation is that because the PHDA was based on the 2011 Census, it excluded people living in England in 2011 but not taking part in the 2011 Census; respondents who could not be linked to the 2011–2013 NHS Patient Register and recent migrants. Consequently, we excluded 5.4% of vaccinated people who could not be linked to the ONS PHDA.

## Conclusion

There are stark differences in COVID-19 vaccination rates by ethnic group and religious affiliation. Research is now urgently needed to understand why these disparities exist in these groups and how they can best be addressed through public health policy and community engagement. Understanding barriers and supporting participation in the vaccine programme is especially important because the groups with low vaccination coverage were also at elevated risk of COVID-19 mortality in the first two waves of the pandemic,^11–14^ are associated with factors, such as frailty, that will continue to elevate risk as the pandemic evolves.^15^

## Supplementary Material

Reviewer comments

Author's manuscript

## Data Availability

Data are not yet available, but will be made available on the ONS Secure Research Service for accredited researchers.

## References

[1] UK Coronavirus Dashboard. Coronavirus (COVID-19) in the UK - vaccinations, 2021. Available: https://coronavirus.data.gov.uk/details/vaccinations

[2] Pebody RG, Hippisley-Cox J, Harcourt S, et al. Uptake of pneumococcal polysaccharide vaccine in at-risk populations in England and Wales 1999-2005. Epidemiol Infect 2008;136:360–9. 10.1017/S0950268807008436 17445314 PMC2870812

[3] Spencer AM, Roberts SA, Brabin L, et al. Sociodemographic factors predicting mother's cervical screening and daughter's HPV vaccination uptake. J Epidemiol Community Health 2014;68:571–7. 10.1136/jech-2013-202629 24567443

[4] Hungerford D, Macpherson P, Farmer S, et al. Effect of socioeconomic deprivation on uptake of measles, mumps and rubella vaccination in Liverpool, UK over 16 years: a longitudinal ecological study. Epidemiol Infect 2016;144:1201–11. 10.1017/S0950268815002599 26542197

[5] MacKenna B, Curtis HJ, Morton CE. Trends, regional variation, and clinical characteristics of COVID-19 vaccine recipients: a retrospective cohort study in 23.4 million patients using OpenSAFELY. medRxiv 2021.

[6] Department for Communities and Local Government,. The English indices of deprivation 2015, 2015. Available: https://www.gov.uk/government/statistics/english-indices-of-deprivation-2015

[7] Clift AK, Coupland CAC, Keogh RH, et al. Living risk prediction algorithm (QCOVID) for risk of hospital admission and mortality from coronavirus 19 in adults: national derivation and validation cohort study. BMJ 2020;371:m3731. 10.1136/bmj.m3731 33082154 PMC7574532

[8] Tan PS, Patone M, Clift AK. Influenza, shingles and pneumococcal vaccine uptake, offer and refusal in adult populations at high-risk for COVID-19: a UK population-based cohort study. SSRN Electronic Journal 2021.

[9] Lane S, MacDonald NE, Marti M, et al. Vaccine hesitancy around the globe: analysis of three years of WHO/UNICEF joint reporting form data-2015-2017. Vaccine 2018;36:3861–7. 10.1016/j.vaccine.2018.03.063 29605516 PMC5999354

[10] Grabenstein JD. What the world's religions teach, applied to vaccines and immune globulins. Vaccine 2013;31:2011–23. 10.1016/j.vaccine.2013.02.026 23499565

[11] Gaughan CH, Ayoubkhani D, Nafilyan V, et al. Religious affiliation and COVID-19-related mortality: a retrospective cohort study of prelockdown and postlockdown risks in England and Wales. J Epidemiol Community Health 2021. 10.1136/jech-2020-215694. [Epub ahead of print: 06 Jan 2021]. 33408165

[12] Ayoubkhani D, Nafilyan V, White C, et al. Ethnic-minority groups in England and Wales-factors associated with the size and timing of elevated COVID-19 mortality: a retrospective cohort study linking census and death records. Int J Epidemiol 2021;49:1951–62. 10.1093/ije/dyaa208 33349855 PMC7799112

[13] Mathur R, Rentsch CT, Morton CE. Ethnic differences in COVID-19 infection, hospitalisation, and mortality: an OpenSAFELY analysis of 17 million adults in England. medRxiv 2020:2020.09.22.20198754.

[14] Nafilyan V, Islam N, Mathur R, et al. Ethnic differences in COVID-19 mortality during the first two waves of the coronavirus pandemic: a nationwide cohort study of 29 million adults in England. Eur J Epidemiol 2021. 10.1007/s10654-021-00765-1. [Epub ahead of print: 16 Jun 2021]. PMC8206182 34132940

[15] Palermo S. Covid-19 pandemic: maximizing future vaccination treatments considering aging and frailty. Front Med 2020;7:632. 10.3389/fmed.2020.558835 PMC7530612 33072783

