# Supplementary material for: Sociodemographic inequality in COVID-19 vaccination coverage among elderly adults in England: a national linked data study
Source: BMJ Open. 2021 Jul 23;11(7):e053402. doi: 10.1136/bmjopen-2021-053402 (PMC8313303; doi:10.1136/bmjopen-2021-053402)
Supplement: Author's manuscript [file bmjopen-2021-053402.draft_revisions.pdf]

BMJ Open is committed to open peer review. As part of this commitment we make the peer review history of every article we publish publicly available.

When an article is published we post the peer reviewers' comments and the authors' responses online. We also post the versions of the paper that were used during peer review. These are the versions that the peer review comments apply to.

The versions of the paper that follow are the versions that were submitted during the peer review process. They are not the versions of record or the final published versions. They should not be cited or distributed as the published version of this manuscript.

BMJ Open is an open access journal and the full, final, typeset and author-corrected version of record of the manuscript is available on our site with no access controls, subscription charges or pay-per-view fees (http://bmjopen.bmj.com).

If you have any questions on BMJ Open's open peer review process please email info.bmjopen@bmj.com

BMJ Open

Sociodemographic inequality in COVID-19 vaccination coverage amongst elderly adults in England: a national linked data study

Journal:	*BMJ Open*
Manuscript ID	bmjopen-2021-053402
Article Type:	Original research
Date Submitted by the Author:	13-May-2021
Complete List of Authors:	Nafilyan, Vahe; Office for National Statistics; London School of Hygiene and Tropical Medicine<br>Dolby, Ted; Office for National Statistics<br>Razieh, Cameron; University of Leicester; University Hospitals of Leicester NHS Trust<br>Gaughan, Charlotte; Office for National Statistics, Methodology<br>Morgan, Jasper; Office for National Statistics<br>Ayoubkhani, Daniel; Office for National Statistics, Methodology<br>Walker, Sarah; University of Oxford, Nuffield department of Medicine<br>Khunti, Kamlesh; University of Leicester; Leicester General Hospital<br>Glickman, Myer; Office for National Statistics<br>Yates, Thomas; University of Leicester, Diabetes Research Centre
Keywords:	COVID-19, Infection control < INFECTIOUS DISEASES, Epidemiology < INFECTIOUS DISEASES

SCHOLARONE™
Manuscripts

I, the Submitting Author has the right to grant and does grant on behalf of all authors of the Work (as defined in the below author licence), an exclusive licence and/or a non-exclusive licence for contributions from authors who are: i) UK Crown employees; ii) where BMJ has agreed a CC-BY licence shall apply, and/or iii) in accordance with the terms applicable for US Federal Government officers or employees acting as part of their official duties; on a worldwide, perpetual, irrevocable, royalty-free basis to BMJ Publishing Group Ltd ("BMJ") its licensees and where the relevant Journal is co-owned by BMJ to the co-owners of the Journal, to publish the Work in this journal and any other BMJ products and to exploit all rights, as set out in our licence.

The Submitting Author accepts and understands that any supply made under these terms is made by BMJ to the Submitting Author unless you are acting as an employee on behalf of your employer or a postgraduate student of an affiliated institution which is paying any applicable article publishing charge ("APC") for Open Access articles. Where the Submitting Author wishes to make the Work available on an Open Access basis (and intends to pay the relevant APC), the terms of reuse of such Open Access shall be governed by a Creative Commons licence – details of these licences and which Creative Commons licence will apply to this Work are set out in our licence referred to above.

Other than as permitted in any relevant BMJ Author's Self Archiving Policies, I confirm this Work has not been accepted for publication elsewhere, is not being considered for publication elsewhere and does not duplicate material already published. I confirm all authors consent to publication of this Work and authorise the granting of this licence.

For peer review only

Sociodemographic inequality in COVID-19 vaccination coverage amongst elderly adults in England: a national linked data study

Vahé Nafilyan[1,2], Ted Dolby[2], Cameron Razieh[3,4] , Charlotte Gaughan[2], Jasper Morgan[2], Daniel Ayoubkhani[2], A. Sarah Walker[5], Kamlesh Khunti[3,4], Myer Glickman[2], Thomas Yates[3,4]

[1] Faculty of Public Health, Environment and Society, London School of Hygiene and Tropical Medicine, London, UK

[2] Office for National Statistics, Newport, UK

[3] Diabetes Research Centre, University of Leicester, Leicester, UK

[4] NIHR Leicester Biomedical Research Centre, University of Leicester and University Hospitals of Leicester NHS Trust, Leicester, UK

[5]Nuffield department of Medicine, University of Oxford, Oxford, UK

Corresponding author:

Vahé Nafilyan (vahe.nafilyan@ons.gov.uk)

For peer review only

Abstract

Objective: To examine inequalities in COVID-19 vaccination rates amongst elderly adults in England

Design: Cohort study

Setting: People living in private households and communal establishments in England

Participants: 6,829,643 adults aged ≥70 years (mean 78.7 years, 55.2% female) who were alive on 15 March 2021.

Main outcome measures: Having received the first dose of a vaccine against COVID-19 by 15 March 2021. We calculated vaccination rates and estimated unadjusted and adjusted odds ratios using logistic regression models.

Results: By 15 March 2021, 93.2% of people living in England aged 70 years and over had received at least one dose of a COVID-19 vaccine. While vaccination rates differed across all factors considered apart from sex, the greatest disparities were seen between ethnic and religious groups. The lowest rates were in people of Black African and Black Caribbean ethnic backgrounds, where only 67.2% and 73.9% had received a vaccine, with adjusted odds of not being vaccinated at 5.01 (95% CI 4.86 - 5.16) and 4.85 (4.75 - 4.96) times greater than the White British group. The proportion of individuals self-identifying as Muslim and Buddhist who had received a vaccine was 79.1% and 84.1%, respectively. Older age, greater area deprivation, less advantaged socio-economic position (proxied by living in a rented home), being disabled and living either alone or in a multi-generational household were also associated with higher odds of not having received the vaccine.

Conclusion: People disproportionately affected seem most hesitant to COVID-19 vaccinations. Policy Interventions to improve these disparities are urgently needed.

For peer review only

Summary Box

What is already known on this subject?

The UK began an ambitious vaccination programme to combat the COVID-19 pandemic on 8th December 2020. Existing evidence suggests that COVID-19 vaccination rates differ by level of area deprivation, ethnicity and certain underlying health conditions, such as learning disability and mental health problems.

What does this study add?

Our study shows that first dose vaccination rates in adults aged 70 or over differed markedly by ethnic group and self-reported religious affiliation, even after adjusting for geography, socio-demographic factors and underlying health conditions. Our study also highlights differences in vaccination rates by deprivation, household composition, and disability status, factors disproportionately associated with SARS-CoV-2 infection. Public health policy and community engagement aimed at promoting vaccination uptake is these groups are urgently needed.

Strengths and limitations of this study

- Using nationwide linked population-level data from clinical records and the 2011 Census, we examined a wide range of socio-demographic characteristics not available n electronic health records
- Most demographic and socio-economic characteristics are derived from the 2011 Census and therefore are 10 years old. However, we focus primarily on characteristics that are unlikely to change over time, such as ethnicity or religion, or likely to be stable for our population
- Because the data are based on the 2011 Census, it excluded people living in England in 2011 but not taking part in the 2011 Census; respondents who could not be linked to the 2011-2013 NHS patients register; recent migrants. Consequently, we excluded 5.4% of vaccinated people who could not be linked

For peer review only

Introduction

The UK began an ambitious vaccination programme to combat the COVID-19 pandemic on 8th December 2020; by 24th April 2021, 64% of the UK adult population have received their first of the dose [1].

Previous research demonstrates that vaccination rates tend to be lower amongst certain ethnic groups, and in areas of higher deprivation [2, 3, 4]. Existing evidence suggests that COVID-19 vaccination rates differ by level of area deprivation, certain underlying health conditions and ethnicity [5]. Far less is known about how COVID-19 vaccination uptake varies by socio-demographic factors, such as religious affiliation, individual socio-economic status, living in multigenerational household or disability status, factors disproportionately associated with SARS-CoV-2 infection. Understanding which socio-demographic, economic, and cultural factors are associated with low vaccination rates has major implications for designing policies that help maximise the vaccination campaign coverage.

This study investigates inequality in vaccination rates amongst adults aged ≥70 years in England, using population-level administrative records linked to the 2011 Census. This enables examination of a wide range of socio-demographic characteristics, currently lacking in previously published studies, in particular ethnicity, religion, different measures of socio-economic position, and those who report being disabled.

Methods

We linked vaccination data from the National Immunisation Management System (NIMS) to the Office for National Statistics (ONS) Public Health Data Asset (PHDA) based on NHS number. The ONS PHDA is a linked dataset combining the 2011 Census, mortality records, the General Practice Extraction Service (GPES) data for pandemic planning and research and the Hospital Episode Statistics (HES). To obtain NHS numbers for the 2011 Census, we linked the 2011 Census to the 2011-2013 NHS Patient Registers using deterministic and probabilistic matching, with an overall linkage rate of 94.6%. All subsequent linkages were performed based on NHS numbers.

The study population consisted of people aged ≥70 years, alive on 15th March 2020, who were resident in England, registered with a general practitioner, and enumerated at the 2011 Census. Of 6,605,315 adults aged ≥70 years who received a first dose of a COVID-19 vaccine in NIMS, 6,242,384 (94.5%) were linked to the ONS PHDA.

The main outcome was having received at least a first dose of a COVID-19 vaccine by 15th March 2021, as recorded in the NIMS data available on 31st March 2021. Phase 1 of the vaccination policy for England aimed to offer a first vaccination appointment to all those ≥70 years by 15th February, and we allowed a further month to ensure full coverage.

This dataset combines comprehensive socio-demographic information from the 2011 Census with a detailed medical history from clinical records. All individual-level socio-demographic characteristics (ethnic group, religious affiliation, disability status, educational attainment) came from the 2011 Census. We used a 10-category ethnic group classification (white British, Bangladeshi, black African, black Caribbean, Chinese, Indian, Mixed, Other, Pakistani, white other). Self-reported religious group, place of residence (region within England, private or care home) and area-based deprivation (Index of Multiple Deprivation [6]) were derived based on the 2019 Patient Register. Comorbidities were defined as in the

For peer review only

QCOVID risk prediction model [7]. All variables included in this analysis are listed in Supplementary Table S1.

First, we estimated the first dose vaccination rates by a range of demographic and socio-economic characteristics. Second, to understand the drivers of the observed differences in vaccination rates, we used logistic regression to estimate the odds of not having received a first dose of a COVID-19 vaccine. For each exposure, we compared odds ratios from an unadjusted model, a model only adjusted for sex and age, and a model further adjusted for all geographic and socio-demographic characteristics, disability status and pre-existing conditions.

Patient and Public Involvement

No patient involved

Results

Our study population included 6,724,179 adults aged ≥70 years who lived in England. 55.1% were women and the mean age was 78.8 (SD: 6.5) years; 91.6% identified themselves as white British, 78.5% as Christian. 82.5% owned their home (Table 1). By 15 March 2021, 93.2% of people living in England aged 70 years and over had received at least one dose of a COVID-19 vaccine.

[Table 1]

Table 2 shows vaccination rates by demographic and socio-economic factors, as well as odds ratios (OR) from different models. Vaccination rates differed across all factors considered, apart from sex. The lowest rates were in people of Black African and Black Caribbean ethnic backgrounds where only 67.2% and 73.9% had received a vaccine. Adjusting for differences in geography, socio-demographic factors and underlying health conditions did not fully explain the lower probability of having received the vaccine among ethnic minority groups. Compared with people of white British ethnicity, the fully adjusted OR for black African individuals was 5.01 [95% CI 4.86 - 5.16], whilst the unadjusted OR was 7.62 [7.40 - 7.84], suggesting that geography, socio-demographic factors and pre-pandemic health only explains about 40% of the elevated odds of not being vaccinated.

Vaccination rates also varied markedly across religious groups. Whilst 93.8% of Christians had been vaccinated, only 79.1% of Muslims and 84.1% of Buddhists had been vaccinated. Stark differences remained after adjustment for other factors, with an adjusted OR of not being vaccinated of 2.74 [2.69 - 2.79] for Muslims and 2.03 [1.95 - 2.12] for Buddhist, compared to Christians.

Greater area deprivation, less advantaged socio-economic position (proxied by living in a rented home), being disabled and living either alone or in a multi-generational household were also associated with low vaccination rates, even when adjusting for other factors (Table 2). These differences were less pronounced than the differences between ethnic groups or religious affiliations.

[Table 2]

For peer review only

Discussion

Main findings

Our analysis using whole population level linked data in England suggests that first dose vaccination rates in adults aged ≥70 differed markedly by ethnic group and self-reported religious affiliation. The percentage of people vaccinated was lower among all minority ethnic groups compared to the White British population, with the lowest vaccination rates observed among Black African, Black Caribbean, Bangladeshi and Pakistani individuals. In addition, lower vaccination rates were reported among individuals who identified as Muslim and Buddhist. Whilst some differences were found by deprivation, household factors, disability status and other socio-demographic factors, these were less pronounced compared with ethnicity or religious affiliation.

Comparison with other studies

Few studies have investigated how COVID-19 vaccination coverage varies by a wide range of socio-demographic characteristics. Our results on ethnicity and area deprivation are consistent with one previous study based on clinical records for 40% of patients in England [3]. In addition, our results confirm studies showing that influenza, shingles and pneumococcal vaccination are patterned by similar factors, including ethnicity, deprivation and household size [8]. Pre-pandemic, religion and culture have been postulated to be important factors in determining vaccination uptake [9]; our results extend this by showing that self-reported religious affiliation is an important factor in COVID-19 vaccine uptake. Differences in vaccination rate and potential vaccination hesitancy between religious groups may not be based upon religious beliefs, but rather reflect safety and other concerns [10], or, given high infection rates in some of these groups [11], beliefs that vaccination is not needed after natural infection. We also find that vaccination rates vary by individual characteristics not reported in previous studies, such as household tenure (a proxy for socio-economic status), household composition and disability status.

Strengths and limitation

The primary study strength is using nationwide linked population-level data from clinical records and the 2011 Census. Unlike studies based solely on electronic health records, we examined a wide range of socio-demographic characteristics. Unlike surveys, we can precisely estimate vaccination rates and odds ratios for small groups. The main limitation is that most demographic and socio-economic characteristics are derived from the 2011 Census and therefore are 10 years old. However, we focus primarily on characteristics that are unlikely to change over time, such as ethnicity or religion, or likely to be stable for our population (adults aged ≥70), such as household tenure. However, for the characteristics likely to change over time, such as disability status, the time difference may introduce some bias into the estimates, although this would be expected to dilute differences, since we are most likely missing some long-term health conditions. Care home residency and area deprivation were derived from the 2019 Patient register and are therefore not subject to the same biases. Another limitation is that because the Public Health Data Asset was based on the 2011 Census, it excluded people living in England in 2011 but not taking part in the 2011 Census; respondents who could not be linked to the 2011-2013 NHS patients register; recent migrants. Consequently, we excluded 5.4% of vaccinated people who could not be linked to the ONS PHDA.

For peer review only

Conclusion

As ethnicity and religion may be the most important determinants of COVID-19 vaccination rates in England, research is now urgently needed to understand why these disparities exist in these groups and how they can best be addressed through public health policy and community engagement. This is especially important because the groups with low vaccination coverage were also at elevated risk of COVID-19 mortality in the first two waves of the pandemic [12, 11, 13, 14].

References

[1] UK Coronavirus Dashboard, "Coronavirus (COVID-19) in the UK - Vaccinations," [Online]. Available: https://coronavirus.data.gov.uk/details/vaccinations. [Accessed 27 04 2021].

[2] R. G. Pebody, J. Hippisley-Cox, S. Harcourt, M. Pringle, M. Painter and G. Smith, "Uptake of pneumococcal polysaccharide vaccine in at-risk populations in England and Wales 1999-2005," *Epidemiology and Infection,* vol. 136, p. 360–369, 3 2008.

[3] A. M. Spencer, S. A. Roberts, L. Brabin, J. Patnick and A. Verma, "Sociodemographic factors predicting mother's cervical screening and daughter's HPV vaccination uptake," *Journal of Epidemiology and Community Health,* vol. 68, p. 571–577, 2014.

[4] D. Hungerford, P. Macpherson, S. Farmer, S. Ghebrehewet, D. Seddon, R. Vivancos and A. Keenan, "Effect of socioeconomic deprivation on uptake of measles, mumps and rubella vaccination in Liverpool, UK over 16 years: A longitudinal ecological study," *Epidemiology and Infection,* vol. 144, p. 1201–1211, 4 2016.

[5] B. MacKenna, H. J. Curtis, C. E. Morton, P. Inglesby, A. J. Walker, J. Morley, A. Mehrkar, S. Bacon, G. Hickman, C. Bates, R. Croker, D. Evans, T. Ward, J. Cockburn, S. Davy, K. Bhaskaran, A. Schultze, C. T. Rentsch, E. Williamson, W. Hulme, H. I. McDonald, L. Tomlinson, R. Mathur, H. Drysdale, R. M. Eggo, K. Wing, A. Y. S. Wong, H. Forbes, J. Parry, F. Hester, S. Harper, I. J. Douglas, S. J. W. Evans, L. Smeeth and B. Goldacre, "Trends, regional variation, and clinical characteristics of COVID-19 vaccine recipients: a retrospective cohort study in 23.4 million patients using OpenSAFELY," *medRxiv,* 2021.

[6] Department for Communities and Local Government, "The English Indices of Deprivation 2015," 2015. [Online]. Available: https://www.gov.uk/government/statistics/english-indices-of-deprivation-2015. [Accessed 3 May 2021].

[7] A. K. Clift, C. A. C. Coupland, R. H. Keogh, K. Diaz-Ordaz, E. Williamson, E. M. Harrison, A. Hayward, H. Hemingway, P. Horby, N. Mehta, J. Benger, K. Khunti, D. Spiegelhalter, A. Sheikh, J. Valabhji, R. A. Lyons, J. Robson, M. G. Semple, F. Kee, P. Johnson, S. Jebb, T. Williams and J. Hippisley-Cox, "Living risk prediction algorithm (QCOVID) for risk of hospital admission and mortality from coronavirus 19 in adults: national derivation and validation cohort study," *BMJ,* vol. 371, 2020.

For peer review only

[8] P. S. Tan, M. Patone, A. K. Clift, H. Dambha-Miller, D. Saatci, T. Ranger, C. Garriga, F. Zaccardi, B. R. Shah, C. Coupland, S. Griffin, K. Khunti and J. Hippisley-Cox, "Influenza, Shingles and Pneumococcal Vaccine Uptake, Offer and Refusal in Adult Populations at High-Risk for COVID-19: A UK Population-Based Cohort Study," *SSRN Electronic Journal,* 2021.

[9] S. Lane, N. E. MacDonald, M. Marti and L. Dumolard, "Vaccine hesitancy around the globe: Analysis of three years of WHO/UNICEF Joint Reporting Form data-2015–2017," *Vaccine,* vol. 36, no. 26, pp. 3861-3867, 2018.

[10] J. D. Grabenstein, "What the World's religions teach, applied to vaccines and immune globulins," *Vaccine,* vol. 31, no. 16, pp. 2011-2023, 2013.

[11] C. H. Gaughan, D. Ayoubkhani, V. Nafilyan, P. Goldblatt, C. White, K. Tingay and N. Bannister, "Religious affiliation and COVID-19-related mortality: A retrospective cohort study of prelockdown and postlockdown risks in England and Wales," *Journal of Epidemiology and Community Health,* vol. 0, p. 1–6, 1 2021.

[12] D. Ayoubkhani, V. Nafilyan, C. White, P. Goldblatt, C. Gaughan, L. Blackwell, N. Rogers, A. Banerjee, K. Khunti, M. Glickman, B. Humberstone and I. Diamond, "Ethnic-minority groups in England and Wales—factors associated with the size and timing of elevated COVID-19 mortality: a retrospective cohort study linking census and death records," *International Journal of Epidemiology,* vol. 49, p. 1951–1962, 1 2021.

[13] R. Mathur, C. T. Rentsch, C. E. Morton, W. J. Hulme, A. Schultze, B. MacKenna, R. Eggo, K. Bhaskaran, A. Y. S. Wong, E. J. Williamson, H. Forbes, K. Wing, H. I. McDonald, C. Bates, S. Bacon, A. J. Walker, D. Evans, P. Inglesby, A. Mehrkar, H. J. Curtis, N. J. DeVito, R. Croker, H. Drysdale, J. Cockburn, J. Parry, F. Hester, S. Harper, I. J. Douglas, L. Tomlinson, S. J. W. Evans, R. Grieve, D. Harrison, K. Rowan, K. Khunti, N. Chaturvedi, L. Smeeth and B. Goldacre, "Ethnic differences in COVID-19 infection, hospitalisation, and mortality: an OpenSAFELY analysis of 17 million adults in England," *medRxiv,* p. 2020.09.22.20198754, 2020.

[14] V. Nafilyan, N. Islam, R. Mathur, D. Ayoubkhani, A. Banerjee, M. Glickman, B. Humberstone, I. Diamond and K. Khunti, "Ethnic differences in COVID-19 mortality during the first two waves of the Coronavirus Pandemic: a nationwide cohort study of 29 million adults in England," *MedRxiv,* 2021.

For peer review only

Acknowledgements
We are grateful to Charlotte Bermingham for useful discussions about this paper.

Funding
This work was supported by a grant from the UKRI (MRC)-DHSC (NIHR) COVID-19 Rapid Response Rolling Call (MR/V020536/1) and from HDR-UK (HDRUK2020.138). KK, TY and CR are supported by the National Institute for Health Research (NIHR) Applied Research Collaboration East Midlands (ARC EM) and the NIHR Leicester Biomedical Research Centre (BRC). ASW is an NIHR Senior Investigator and is supported by the NIHR Health Protection Research Unit in Healthcare Associated Infections and Antimicrobial Resistance at

Oxford University in partnership with Public Health England (PHE) (NIHR200915) and the NIHR Biomedical Research Centre, Oxford. The views expressed in this publication are those of the authors and not necessarily those of the NHS, the National Institute for Health Research, the Department of Health or Public Health England.

Conflict of interest

KK is Director of the University of Leicester Centre for Black Minority Ethnic Health, Trustee of the South Asian Health Foundation, Chair of the Ethnicity Subgroup of SAGE and Member of Independent SAGE**.**

For peer review only

Tables

Table 1: Characteristics of the study population

Variable	Level	Count (%)
Vaccinated		6,202,780 (93.20)
Sex	Female	3,672,314 (55.18)
	Male	2,983,358 (44.82)
Age	Mean (SD)	78.8 (6.49)
Ethnicity	Bangladeshi	11,522 (0.17)
	Black African	21,535 (0.32)
	Black Caribbean	52,883 (0.79)
	Chinese	18,452 (0.28)
	Indian	103,564 (1.56)
	Mixed	24,637 (0.37)
	Other	65,241 (0.98)
	Pakistani	39,723 (0.60)
	White British	6,095,276 (91.58)
	White other	222,839 (3.35)
Religion	Buddhist	16,403 (0.25)
	Christian	5,221,392 (78.45)
	Hindu	61,634 (0.93)
	Jewish	39,800 (0.60)
	Muslim	86,841 (1.30)
	No religion	725,695 (10.90)
	Other Religion	22,327 (0.34)
	Religion Not Stated	449,781 (6.76)
	Sikh	31,799 (0.48)
IMD Quintile	1 (Most deprived)	913,809 (13.73)
	2	1,140,651 (17.14)
	3	1,407,155 (21.14)
	4	1,560,023 (23.44)
	5 (Least deprived)	1,634,034 (24.55)
Household tenure	Owned	5,488,126 (82.46)
	Other	114,972 (1.73)
	Private rented	273,707 (4.11)
	Social rented	778,867 (11.70)
Rural-Urban	Rural	6,005,144 (82.43)
	Urban	304,412 (4.18)
Household composition	2 Elderly	847,508 (11.63)
	1 Elderly	128,083 (1.76)

only

Care home	436,211 (5.99)
Missing household	9,035 (0.14)
Multigenerational	620,167 (8.51)
Other (3+ Adults)	714,211 (9.80)

Note: Adults aged 70 years or over, living in England, alive on 15th March 2021.

For peer review only

Table 2

Exposure	Group	Vaccination rate	Odds ratio (Model 1)	Odds ratio (Model 2)	Odds ratio (Model 3)
Age group	70-74	90.9 [90.8 - 90.9]	1 (Ref)	1 (Ref)	1 (Ref)
	75-79	93.8 [93.8 - 93.9]	0.65 [0.65 - 0.66]	0.65 [0.65 - 0.66]	0.66 [0.65 - 0.66]
	80-84	95.6 [95.5 - 95.6]	0.46 [0.46 - 0.46]	0.46 [0.46 - 0.46]	0.45 [0.45 - 0.46]
	85-89	95.1 [95.0 - 95.1]	0.51 [0.51 - 0.52]	0.51 [0.51 - 0.52]	0.51 [0.51 - 0.52]
	90-94	94.0 [93.9 - 94.1]	0.63 [0.62 - 0.64]	0.63 [0.62 - 0.64]	0.64 [0.63 - 0.65]
	95-99	92.3 [92.1 - 92.5]	0.83 [0.81 - 0.85]	0.82 [0.80 - 0.85]	0.82 [0.80 - 0.84]
	100+	85.5 [84.8 - 86.1]	1.69 [1.61 - 1.78]	1.68 [1.60 - 1.77]	1.60 [1.52 - 1.68]
Sex	Female	93.2 [93.2 - 93.2]	1 (Ref)	1 (Ref)	1 (Ref)
	Male	93.2 [93.2 - 93.2]	1.00 [1.00 - 1.01]	0.98 [0.98 - 0.99]	1.03 [1.02 - 1.03]
Disability	Not Limited	93.5 [93.5 - 93.5]	1 (Ref)	1 (Ref)	1 (Ref)
	Limited a little	93.4 [93.3 - 93.4]	1.02 [1.01 - 1.03]	1.12 [1.11 - 1.13]	1.08 [1.07 - 1.08]
	Limited a lot	91.3 [91.2 - 91.3]	1.37 [1.36 - 1.38]	1.47 [1.46 - 1.48]	1.29 [1.28 - 1.30]
Ethnicity	White British	94.0 [94.0 - 94.0]	1 (Ref)	1 (Ref)	1 (Ref)
	Bangladeshi	82.7 [82.0 - 83.4]	3.26 [3.11 - 3.42]	3.48 [3.31 - 3.65]	2.56 [2.43 - 2.69]
	Black African	67.2 [66.6 - 67.8]	7.62 [7.40 - 7.84]	7.59 [7.37 - 7.81]	5.01 [4.86 - 5.16]
	Black Caribbean	73.8 [73.4 - 74.2]	5.55 [5.44 - 5.66]	6.35 [6.22 - 6.47]	4.85 [4.75 - 4.96]
	Chinese	82.8 [82.3 - 83.4]	3.23 [3.11 - 3.36]	3.11 [2.99 - 3.23]	2.64 [2.54 - 2.75]
	Indian	90.9 [90.7 - 91.0]	1.57 [1.54 - 1.60]	1.55 [1.52 - 1.59]	1.35 [1.32 - 1.38]
	Mixed	85.3 [84.9 - 85.7]	2.69 [2.60 - 2.79]	2.67 [2.58 - 2.77]	2.21 [2.14 - 2.30]
	Other	82.9 [82.6 - 83.2]	3.22 [3.15 - 3.29]	3.12 [3.05 - 3.18]	2.44 [2.39 - 2.50]
	Pakistani	79.6 [79.2 - 80.0]	3.99 [3.89 - 4.09]	4.12 [4.02 - 4.22]	3.59 [3.50 - 3.68]
	White other	87.7 [87.5 - 87.8]	2.20 [2.17 - 2.23]	2.24 [2.21 - 2.27]	1.93 [1.90 - 1.95]
IMD Quintile	1 (most deprived)	90.6 [90.6 - 90.7]	1.77 [1.76 - 1.79]	1.76 [1.75 - 1.78]	1.60 [1.59 - 1.62]
	2	92.1 [92.0 - 92.1]	1.48 [1.46 - 1.49]	1.47 [1.46 - 1.48]	1.34 [1.33 - 1.35]
	3	93.4 [93.4 - 93.5]	1.20 [1.19 - 1.22]	1.20 [1.19 - 1.21]	1.17 [1.16 - 1.18]
	4	94.0 [94.0 - 94.0]	1.09 [1.08 - 1.10]	1.09 [1.08 - 1.10]	1.09 [1.08 - 1.10]
	5 (least deprived)	94.5 [94.4 - 94.5]	1 (Ref)	1 (Ref)	1 (Ref)
Religion	Christian	93.8 [93.8 - 93.9]	1 (Ref)	1 (Ref)	1 (Ref)
	Buddhist	84.1 [83.5 - 84.7]	2.88 [2.76 - 3.01]	2.63 [2.52 - 2.74]	2.03 [1.95 - 2.12]
	Hindu	91.5 [91.2 - 91.7]	1.43 [1.39 - 1.47]	1.39 [1.35 - 1.43]	1.03 [1.00 - 1.06]
	Jewish	93.1 [92.8 - 93.3]	1.13 [1.09 - 1.18]	1.12 [1.08 - 1.17]	0.94 [0.90 - 0.97]
	Muslim	79.1 [78.9 - 79.4]	4.02 [3.95 - 4.09]	4.04 [3.97 - 4.11]	2.74 [2.69 - 2.79]
	No religion	91.9 [91.9 - 92.0]	1.34 [1.33 - 1.35]	1.25 [1.23 - 1.26]	1.23 [1.22 - 1.24]
	Other Religion	85.4 [84.9 - 85.8]	2.61 [2.51 - 2.71]	2.41 [2.32 - 2.50]	2.15 [2.07 - 2.23]
	Religion Not Stated	91.5 [91.4 - 91.6]	1.42 [1.41 - 1.44]	1.40 [1.38 - 1.41]	1.35 [1.33 - 1.36]
	Sikh	91.6 [91.3 - 91.9]	1.39 [1.34 - 1.45]	1.35 [1.30 - 1.41]	1.07 [1.03 - 1.11]
Household tenure	Owned	94.0 [93.9 - 94.0]	1 (Ref)	1 (Ref)	1 (Ref)
	Other	91.1 [90.9 - 91.2]	1.52 [1.49 - 1.55]	1.55 [1.52 - 1.58]	1.49 [1.46 - 1.52]

	Private rented	88.4 [88.2 - 88.5]	2.05 [2.02 - 2.07]	1.96 [1.94 - 1.99]	1.81 [1.79 - 1.83]
	Social rented	89.9 [89.8 - 89.9]	1.75 [1.73 - 1.76]	1.77 [1.76 - 1.79]	1.60 [1.59 - 1.61]
Rural-Urban	Rural	94.5 [94.4 - 94.5]	1 (Ref)	1 (Ref)	1 (Ref)
	Urban	92.8 [92.8 - 92.8]	1.33 [1.32 - 1.34]	1.34 [1.33 - 1.35]	1.12 [1.12 - 1.13]
Household composition	2 Elderly	94.5 [94.4 - 94.5]	1 (Ref)	1 (Ref)	1 (Ref)
	1 Elderly	92.5 [92.5 - 92.6]	1.38 [1.37 - 1.39]	1.53 [1.52 - 1.54]	1.32 [1.31 - 1.33]
	Care home	94.9 [94.8 - 95.0]	0.92 [0.89 - 0.95]	1.10 [1.06 - 1.13]	0.89 [0.86 - 0.91]
	Multigenerational	90.3 [90.3 - 90.4]	1.83 [1.82 - 1.85]	1.77 [1.76 - 1.79]	1.39 [1.38 - 1.40]
	Other (3+ Adults)	90.0 [89.6 - 90.3]	1.91 [1.84 - 1.97]	1.81 [1.75 - 1.88]	1.54 [1.49 - 1.60]

Note: Adults aged 70 years or over, living in England, alive on 15th March 2021. Model 1: unadjusted; Model 2: adjusted for sex and age (Cubic splines); Model 3: adjusted for sex, age (Cubic splines), care home status, rural/urban, region, ethnicity (except when looking at religion as an exposure), IMD quintile (except when looking at household tenure as an exposure), disability, BMI and comorbidities. See Supplementary Table S1 for more details on the variables included in the models.

For peer review only

Sociodemographic inequality in COVID-19 vaccination coverage amongst elderly adults in England: a national linked data study – supplementary material

Appendix

Supplementary Table 1: Variables used in the analyses

Variable	Coding	Source
Vaccinated	Received a first dose of a COVID-19 vaccine by 15th March 2020	NIMS
Age	Third-order polynomial	2011 Census
Sex	Female, Male	2011 Census
Ethnicity	White British, Bangladeshi, Black African, Black Caribbean, Chinese, Indian, Mixed, Other, Pakistani, White other	2011 Census
Religious affiliation	Christian, Buddhist, Hindu, Jewish, Muslim, No religion, Other Religion, Religion Not Stated, Sikh	2011 Census
Region	Dummy variables representing region of residence	2019 NHS PR
Rural urban classification	Urban, rural	2019 NHS PR
Index of Multiple Deprivation (IMD)	Dummy variables representing quintiles of deprivation	2019 NHS PR
Household tenure	Own, social rented, private rented, other	2011 Census
Level of highest qualification	Degree, A-level or equivalent, GCSE or equivalent, no qualification, other	2011 Census
Disability	Non-disabled , disabled (limited a little), disabled (limited a lot)	2011 Census
Body Mass Index (kg/m2)	< 18.5, 18.5 – 25, 25 to 30, >= 30, missing	GPES
Chronic kidney disease (CKD)	No CKD, CKD3, CKD4, CKD5	GPES
Learning disability	No learning disability, Down's Syndrome, other learning disability	GPES
Cancer and immunosuppression	Dummies for blood cancer, solid organ transplant , Prescribed immunosuppressant medication by GP , Prescribed leukotriene or long-acting beta blockers, Prescribed regular prednisolone ,	GPES

For peer review only

Other conditions	Diabetes, Chronic obstructive pulmonary disease (COPD), Asthma , Rare pulmonary diseases , Pulmonary hypertension or pulmonary fibrosis , Coronary heart disease , Stroke , Atrial Fibrillation , Congestive cardiac failure , Venous thromboembolism , Peripheral vascular disease , Congenital heart disease , Dementia , Parkinson's disease , Epilepsy , Rare neurological conditions , Cerebral palsy , Severe mental illness (bipolar disorder, schizophrenia, severe depression), Osteoporotic fracture , Rheumatoid arthritis or Systemic lupus erythematosus , Cirrhosis of the liver	GPES/HES

For peer review only

STROBE Statement—Checklist of items that should be included in reports of ***cohort studies***

	Item No	Recommendation	Page No
**Title and abstract**	1	(*a*) Indicate the study's design with a commonly used term in the title or the abstract	1
		(*b*) Provide in the abstract an informative and balanced summary of what was done and what was found	2
**Introduction**			
Background/rationale	2	Explain the scientific background and rationale for the investigation being reported	3
Objectives	3	State specific objectives, including any prespecified hypotheses	3
**Methods**			
Study design	4	Present key elements of study design early in the paper	3
Setting	5	Describe the setting, locations, and relevant dates, including periods of recruitment, exposure, follow-up, and data collection	3
Participants	6	(*a*) Give the eligibility criteria, and the sources and methods of selection of participants. Describe methods of follow-up	3
		(*b*) For matched studies, give matching criteria and number of exposed and unexposed	
Variables	7	Clearly define all outcomes, exposures, predictors, potential confounders, and effect modifiers. Give diagnostic criteria, if applicable	3
Data sources/ measurement	8*	For each variable of interest, give sources of data and details of methods of assessment (measurement). Describe comparability of assessment methods if there is more than one group	3
Bias	9	Describe any efforts to address potential sources of bias	3
Study size	10	Explain how the study size was arrived at	3
Quantitative variables	11	Explain how quantitative variables were handled in the analyses. If applicable, describe which groupings were chosen and why	4 Table S1
Statistical methods	12	(*a*) Describe all statistical methods, including those used to control for confounding	4
		(*b*) Describe any methods used to examine subgroups and interactions	NA
		(*c*) Explain how missing data were addressed	NA
		(*d*) If applicable, explain how loss to follow-up was addressed	NA
		(*e*) Describe any sensitivity analyses	NA
**Results**			
Participants	13*	(a) Report numbers of individuals at each stage of study—eg numbers potentially eligible, examined for eligibility, confirmed eligible, included in the study, completing follow-up, and analysed	3,4
		(b) Give reasons for non-participation at each stage	3
		(c) Consider use of a flow diagram	
Descriptive data	14*	(a) Give characteristics of study participants (eg demographic, clinical, social) and information on exposures and potential confounders	4 (Table1)
		(b) Indicate number of participants with missing data for each variable of interest	4 (Table 1)
		(c) Summarise follow-up time (eg, average and total amount)	
Outcome data	15*	Report numbers of outcome events or summary measures over time	Table 1

For peer review only

For peer review only - http://bmjopen.bmj.com/site/about/guidelines.xhtml

Main results	16	(*a*) Give unadjusted estimates and, if applicable, confounder-adjusted estimates and their precision (eg, 95% confidence interval). Make clear which confounders were adjusted for and why they were included<br>(*b*) Report category boundaries when continuous variables were categorized<br>(*c*) If relevant, consider translating estimates of relative risk into absolute risk for a meaningful time period	4
Other analyses	17	Report other analyses done—eg analyses of subgroups and interactions, and sensitivity analyses	
**Discussion**			
Key results	18	Summarise key results with reference to study objectives	5
Limitations	19	Discuss limitations of the study, taking into account sources of potential bias or imprecision. Discuss both direction and magnitude of any potential bias	5
Interpretation	20	Give a cautious overall interpretation of results considering objectives, limitations, multiplicity of analyses, results from similar studies, and other relevant evidence	5
Generalisability	21	Discuss the generalisability (external validity) of the study results	5
**Other information**			
Funding	22	Give the source of funding and the role of the funders for the present study and, if applicable, for the original study on which the present article is based	7

*Give information separately for exposed and unexposed groups.

Note: An Explanation and Elaboration article discusses each checklist item and gives methodological background and published examples of transparent reporting. The STROBE checklist is best used in conjunction with this article (freely available on the Web sites of PLoS Medicine at http://www.plosmedicine.org/, Annals of Internal Medicine at http://www.annals.org/, and Epidemiology at http://www.epidem.com/). Information on the STROBE Initiative is available at http://www.strobe-statement.org.

For peer review only

For peer review only - http://bmjopen.bmj.com/site/about/guidelines.xhtml

BMJ Open

Sociodemographic inequality in COVID-19 vaccination coverage amongst elderly adults in England: a national linked data study

Journal:	*BMJ Open*
Manuscript ID	bmjopen-2021-053402.R1
Article Type:	Original research
Date Submitted by the Author:	23-Jun-2021
Complete List of Authors:	Nafilyan, Vahe; Office for National Statistics; London School of Hygiene and Tropical Medicine<br>Dolby, Ted; Office for National Statistics<br>Razieh, Cameron; University of Leicester; University Hospitals of Leicester NHS Trust<br>Gaughan, Charlotte; Office for National Statistics, Methodology<br>Morgan, Jasper; Office for National Statistics<br>Ayoubkhani, Daniel; Office for National Statistics, Methodology<br>Walker, Sarah; University of Oxford, Nuffield department of Medicine<br>Khunti, Kamlesh; University of Leicester; Leicester General Hospital<br>Glickman, Myer; Office for National Statistics<br>Yates, Thomas; University of Leicester, Diabetes Research Centre
**Primary Subject Heading**:	Public health
Secondary Subject Heading:	Health policy
Keywords:	COVID-19, Infection control < INFECTIOUS DISEASES, Epidemiology < INFECTIOUS DISEASES

SCHOLARONE™
Manuscripts

I, the Submitting Author has the right to grant and does grant on behalf of all authors of the Work (as defined in the below author licence), an exclusive licence and/or a non-exclusive licence for contributions from authors who are: i) UK Crown employees; ii) where BMJ has agreed a CC-BY licence shall apply, and/or iii) in accordance with the terms applicable for US Federal Government officers or employees acting as part of their official duties; on a worldwide, perpetual, irrevocable, royalty-free basis to BMJ Publishing Group Ltd ("BMJ") its licensees and where the relevant Journal is co-owned by BMJ to the co-owners of the Journal, to publish the Work in this journal and any other BMJ products and to exploit all rights, as set out in our licence.

The Submitting Author accepts and understands that any supply made under these terms is made by BMJ to the Submitting Author unless you are acting as an employee on behalf of your employer or a postgraduate student of an affiliated institution which is paying any applicable article publishing charge ("APC") for Open Access articles. Where the Submitting Author wishes to make the Work available on an Open Access basis (and intends to pay the relevant APC), the terms of reuse of such Open Access shall be governed by a Creative Commons licence – details of these licences and which Creative Commons licence will apply to this Work are set out in our licence referred to above.

Other than as permitted in any relevant BMJ Author's Self Archiving Policies, I confirm this Work has not been accepted for publication elsewhere, is not being considered for publication elsewhere and does not duplicate material already published. I confirm all authors consent to publication of this Work and authorise the granting of this licence.

For peer review only

Sociodemographic inequality in COVID-19 vaccination coverage amongst elderly adults in England: a national linked data study

Vahé Nafilyan[1,2], Ted Dolby[2], Cameron Razieh[3,4] , Charlotte Gaughan[2], Jasper Morgan[2], Daniel Ayoubkhani[2], A. Sarah Walker[5], Kamlesh Khunti[3,4], Myer Glickman[2], Thomas Yates[3,4]

[1] Faculty of Public Health, Environment and Society, London School of Hygiene and Tropical Medicine, London, UK

[2] Office for National Statistics, Newport, UK

[3] Diabetes Research Centre, University of Leicester, Leicester, UK

[4] NIHR Leicester Biomedical Research Centre, University of Leicester and University Hospitals of Leicester NHS Trust, Leicester, UK

[5]Nuffield department of Medicine, University of Oxford, Oxford, UK

Corresponding author:

Vahé Nafilyan (vahe.nafilyan@ons.gov.uk)

For peer review only

Abstract

Objective: To examine inequalities in COVID-19 vaccination rates amongst elderly adults in England

Design: Cohort study

Setting: People living in private households and communal establishments in England

Participants: 6,829,643 adults aged ≥70 years (mean 78.7 years, 55.2% female) who were alive on 15 March 2021.

Main outcome measures: Having received the first dose of a vaccine against COVID-19 by 15 March 2021. We calculated vaccination rates and estimated unadjusted and adjusted odds ratios using logistic regression models.

Results: By 15 March 2021, 93.2% of people living in England aged 70 years and over had received at least one dose of a COVID-19 vaccine. While vaccination rates differed across all factors considered apart from sex, the greatest disparities were seen between ethnic and religious groups. The lowest rates were in people of Black African and Black Caribbean ethnic backgrounds, where only 67.2% and 73.9% had received a vaccine, with adjusted odds of not being vaccinated at 5.01 (95% CI 4.86 - 5.16) and 4.85 (4.75 - 4.96) times greater than the White British group. The proportion of individuals self-identifying as Muslim and Buddhist who had received a vaccine was 79.1% and 84.1%, respectively. Older age, greater area deprivation, less advantaged socio-economic position (proxied by living in a rented home), being disabled and living either alone or in a multi-generational household were also associated with higher odds of not having received the vaccine.

Conclusion: Research is now urgently needed to understand why disparities exist in these groups and how they can best be addressed through public health policy and community engagement.

For peer review only

Summary Box

What is already known on this subject?

The UK began an ambitious vaccination programme to combat the COVID-19 pandemic on 8th December 2020. Existing evidence suggests that COVID-19 vaccination rates differ by level of area deprivation, ethnicity and certain underlying health conditions, such as learning disability and mental health problems.

What does this study add?

Our study shows that first dose vaccination rates in adults aged 70 or over differed markedly by ethnic group and self-reported religious affiliation, even after adjusting for geography, socio-demographic factors and underlying health conditions. Our study also highlights differences in vaccination rates by deprivation, household composition, and disability status, factors disproportionately associated with SARS-CoV-2 infection. Public health policy and community engagement aimed at promoting vaccination uptake is these groups are urgently needed.

Strengths and limitations of this study

- Using nationwide linked population-level data from clinical records and the 2011 Census, we examined a wide range of socio-demographic characteristics not available n electronic health records
- Most demographic and socio-economic characteristics are derived from the 2011 Census and therefore are 10 years old. However, we focus primarily on characteristics that are unlikely to change over time, such as ethnicity or religion, or likely to be stable for our population
- Because the data are based on the 2011 Census, it excluded people living in England in 2011 but not taking part in the 2011 Census; respondents who could not be linked to the 2011-2013 NHS patients register; recent migrants. Consequently, we excluded 5.4% of vaccinated people who could not be linked

For peer review only

Introduction

The UK began an ambitious vaccination programme to combat the COVID-19 pandemic on 8th December 2020; by 24th April 2021, 64% of the UK adult population have received their first of the dose [1].

Previous research demonstrates that vaccination rates tend to be lower amongst certain ethnic groups, and in areas of higher deprivation [2, 3, 4]. Existing evidence suggests that COVID-19 vaccination rates differ by level of area deprivation, certain underlying health conditions and ethnicity [5]. Far less is known about how COVID-19 vaccination uptake varies by socio-demographic factors, such as religious affiliation, individual socio-economic status, living in multigenerational household or disability status, factors disproportionately associated with SARS-CoV-2 infection. Understanding which socio-demographic, economic, and cultural factors are associated with low vaccination rates has major implications for designing policies that help maximise the vaccination campaign coverage.

This study investigates inequality in vaccination rates amongst adults aged ≥70 years in England, using population-level administrative records linked to the 2011 Census. This enables examination of a wide range of socio-demographic characteristics, currently lacking in previously published studies, in particular ethnicity, religion, different measures of socio-economic position, and those who report being disabled.

Methods

Study data

We linked vaccination data from the National Immunisation Management System (NIMS) to the Office for National Statistics (ONS) Public Health Data Asset (PHDA) based on NHS number. The ONS PHDA is a linked dataset combining the 2011 Census, mortality records, the General Practice Extraction Service (GPES) data for pandemic planning and research and the Hospital Episode Statistics (HES). To obtain NHS numbers for the 2011 Census, we linked the 2011 Census to the 2011-2013 NHS Patient Registers using deterministic and probabilistic matching, with an overall linkage rate of 94.6%. All subsequent linkages were performed based on NHS numbers.

The study population consisted of people aged ≥70 years, alive on 15th March 2020, who were resident in England, registered with a general practitioner, and enumerated at the 2011 Census. Of 6,605,315 adults aged ≥70 years who received a first dose of a COVID-19 vaccine in NIMS, 6,242,384 (94.5%) were linked to the ONS PHDA.

Outcome

The main outcome was having received at least a first dose of a COVID-19 vaccine by 15th March 2021, as recorded in the NIMS data available on 31st March 2021. Phase 1 of the vaccination policy for England aimed to offer a first vaccination appointment to all those ≥70 years by 15th February, and we allowed a further month to ensure full coverage.

Exposures and covariates

This dataset combines comprehensive socio-demographic information from the 2011 Census with a detailed medical history from clinical records. All individual-level socio-demographic characteristics

For peer review only

(ethnic group, religious affiliation, disability status, educational attainment) came from the 2011 Census. We used a 10-category ethnic group classification (white British, Bangladeshi, black African, black Caribbean, Chinese, Indian, Mixed, Other, Pakistani, white other). Self-reported religious group, place of residence (region within England, private or care home) and area-based deprivation (Index of Multiple Deprivation [6]) were derived based on the 2019 Patient Register. Comorbidities were defined as in the QCOVID risk prediction model, a model used to assess the risk of severe COVID-19 outcomes in the general population, used to inform the prioritisation of the vaccination campaign [7]. All variables included in this analysis are listed in Table 1.

[Table 1]

Statistical analyses

First, we estimated the first dose vaccination rates by a range of demographic and socio-economic characteristics. Second, to understand the drivers of the observed differences in vaccination rates, we used logistic regression to estimate the odds of not having received a first dose of a COVID-19 vaccine. For each exposure, we compared odds ratios (ORs) from models adjusted for different sets of covariates. We estimated unadjusted ORs, ORs adjusted for sex and age, and ORs adjusted for all geographic and socio-demographic characteristics, disability status and pre-existing conditions. All analyses were conducted using R 3.5

Patient and Public Involvement

No patient involved

Results

Our study population included 6,724,179 adults aged ≥70 years who lived in England. 55.1% were women and the mean age was 78.8 (SD: 6.5) years; 91.6% identified themselves as white British, 78.5% as Christian. 82.5% owned their home (Table 2). By 15 March 2021, 93.2% of people living in England aged 70 years and over had received at least one dose of a COVID-19 vaccine.

[Table 2]

Table 3 shows vaccination rates by demographic and socio-economic factors, as well as odds ratios (OR) from different models. Vaccination rates differed across all factors considered, apart from sex. The lowest rates were in people of Black African and Black Caribbean ethnic backgrounds where only 67.2% and 73.9% had received a vaccine. Adjusting for differences in geography, socio-demographic factors and underlying health conditions did not fully explain the lower probability of having received the vaccine among ethnic minority groups. Compared with people of white British ethnicity, the fully adjusted OR for black African individuals was 5.01 [95% CI 4.86 - 5.16], whilst the unadjusted OR was 7.62 [7.40 - 7.84], suggesting that geography, socio-demographic factors and pre-pandemic health only explains about 40% of the elevated odds of not being vaccinated.

Vaccination rates also varied markedly across religious groups. Whilst 93.8% of Christians had been vaccinated, only 79.1% of Muslims and 84.1% of Buddhists had been vaccinated. Stark differences remained after adjustment for other factors, with an adjusted OR of not being vaccinated of 2.74 [2.69 - 2.79] for Muslims and 2.03 [1.95 - 2.12] for Buddhist, compared to Christians.

For peer review only

Greater area deprivation, less advantaged socio-economic position (proxied by living in a rented home), being disabled and living either alone or in a multi-generational household were also associated with low vaccination rates, even when adjusting for other factors (Table 3). These differences were less pronounced than the differences between ethnic groups or religious affiliations.

[Table 3]

Discussion

Main findings

Our analysis using whole population level linked data in England suggests that first dose vaccination rates in adults aged ≥70 differed markedly by ethnic group and self-reported religious affiliation. The percentage of people vaccinated was lower among all minority ethnic groups compared to the White British population, with the lowest vaccination rates observed among Black African, Black Caribbean, Bangladeshi and Pakistani individuals. In addition, lower vaccination rates were reported among individuals who identified as Muslim and Buddhist. Whilst some differences were found by deprivation, household factors, disability status and other socio-demographic factors, these were less pronounced compared with ethnicity or religious affiliation.

Comparison with other studies

Few studies have investigated how COVID-19 vaccination coverage varies by a wide range of socio-demographic characteristics. Our results on ethnicity and area deprivation are consistent with one previous study based on clinical records for 40% of patients in England [3]. In addition, our results confirm studies showing that influenza, shingles and pneumococcal vaccination are patterned by similar factors, including ethnicity, deprivation and household size [8]. Pre-pandemic, religion and culture have been postulated to be important factors in determining vaccination uptake [9]; our results extend this by showing that self-reported religious affiliation is an important factor in COVID-19 vaccine uptake. Differences in vaccination rate and potential vaccination hesitancy between religious groups may not be based upon religious beliefs, but rather reflect safety and other concerns [10], or, given high infection rates in some of these groups [11], beliefs that vaccination is not needed after natural infection. We also find that vaccination rates vary by individual characteristics not reported in previous studies, such as household tenure (a proxy for socio-economic status), household composition and disability status.

Strengths and limitation

The primary study strength is using nationwide linked population-level data from clinical records and the 2011 Census. Unlike studies based solely on electronic health records, we examined a wide range of socio-demographic characteristics. Unlike surveys, we can precisely estimate vaccination rates and odds ratios for small groups. The main limitation is that most demographic and socio-economic characteristics are derived from the 2011 Census and therefore are 10 years old. However, we focus primarily on characteristics that are unlikely to change over time, such as ethnicity or religion, or likely to be stable for our population (adults aged ≥70), such as household tenure. However, for the characteristics likely to change over time, such as disability status, the time difference may introduce some bias into the

For peer review only

estimates, although this would be expected to dilute differences, since we are most likely missing some long-term health conditions. Care home residency and area deprivation were derived from the 2019 Patient register and are therefore not subject to the same biases. Another limitation is that because the Public Health Data Asset was based on the 2011 Census, it excluded people living in England in 2011 but not taking part in the 2011 Census; respondents who could not be linked to the 2011-2013 NHS patients register; recent migrants. Consequently, we excluded 5.4% of vaccinated people who could not be linked to the ONS PHDA.

Conclusion

There are stark differences in COVID-19 vaccination rates by ethnic group and religious affiliation. Research is now urgently needed to understand why these disparities exist in these groups and how they can best be addressed through public health policy and community engagement. Understanding barriers and supporting participation in the vaccine programme is especially important because the groups with low vaccination coverage were also at elevated risk of COVID-19 mortality in the first two waves of the pandemic [12, 11, 13, 14], are associated with factors, such as frailty, that will continue to elevate risk as the pandemic evolves [15]

References

[1] UK Coronavirus Dashboard, "Coronavirus (COVID-19) in the UK - Vaccinations," [Online]. Available: https://coronavirus.data.gov.uk/details/vaccinations. [Accessed 27 04 2021].

[2] R. G. Pebody, J. Hippisley-Cox, S. Harcourt, M. Pringle, M. Painter and G. Smith, "Uptake of pneumococcal polysaccharide vaccine in at-risk populations in England and Wales 1999-2005," *Epidemiology and Infection,* vol. 136, p. 360–369, 3 2008.

[3] A. M. Spencer, S. A. Roberts, L. Brabin, J. Patnick and A. Verma, "Sociodemographic factors predicting mother's cervical screening and daughter's HPV vaccination uptake," *Journal of Epidemiology and Community Health,* vol. 68, p. 571–577, 2014.

[4] D. Hungerford, P. Macpherson, S. Farmer, S. Ghebrehewet, D. Seddon, R. Vivancos and A. Keenan, "Effect of socioeconomic deprivation on uptake of measles, mumps and rubella vaccination in Liverpool, UK over 16 years: A longitudinal ecological study," *Epidemiology and Infection,* vol. 144, p. 1201–1211, 4 2016.

[5] B. MacKenna, H. J. Curtis, C. E. Morton, P. Inglesby, A. J. Walker, J. Morley, A. Mehrkar, S. Bacon, G. Hickman, C. Bates, R. Croker, D. Evans, T. Ward, J. Cockburn, S. Davy, K. Bhaskaran, A. Schultze, C. T. Rentsch, E. Williamson, W. Hulme, H. I. McDonald, L. Tomlinson, R. Mathur, H. Drysdale, R. M. Eggo, K. Wing, A. Y. S. Wong, H. Forbes, J. Parry, F. Hester, S. Harper, I. J. Douglas, S. J. W. Evans, L. Smeeth and B. Goldacre, "Trends, regional variation, and clinical characteristics of COVID-19 vaccine recipients: a retrospective cohort study in 23.4 million patients using OpenSAFELY," *medRxiv,* 2021.

For peer review only

[6] Department for Communities and Local Government, “The English Indices of Deprivation 2015,” 2015. [Online]. Available: https://www.gov.uk/government/statistics/english-indices-of-deprivation-2015. [Accessed 3 May 2021].

[7] A. K. Clift, C. A. C. Coupland, R. H. Keogh, K. Diaz-Ordaz, E. Williamson, E. M. Harrison, A. Hayward, H. Hemingway, P. Horby, N. Mehta, J. Benger, K. Khunti, D. Spiegelhalter, A. Sheikh, J. Valabhji, R. A. Lyons, J. Robson, M. G. Semple, F. Kee, P. Johnson, S. Jebb, T. Williams and J. Hippisley-Cox, “Living risk prediction algorithm (QCOVID) for risk of hospital admission and mortality from coronavirus 19 in adults: national derivation and validation cohort study,” *BMJ,* vol. 371, 2020.

[8] P. S. Tan, M. Patone, A. K. Clift, H. Dambha-Miller, D. Saatci, T. Ranger, C. Garriga, F. Zaccardi, B. R. Shah, C. Coupland, S. Griffin, K. Khunti and J. Hippisley-Cox, “Influenza, Shingles and Pneumococcal Vaccine Uptake, Offer and Refusal in Adult Populations at High-Risk for COVID-19: A UK Population-Based Cohort Study,” *SSRN Electronic Journal,* 2021.

[9] S. Lane, N. E. MacDonald, M. Marti and L. Dumolard, “Vaccine hesitancy around the globe: Analysis of three years of WHO/UNICEF Joint Reporting Form data-2015–2017,” *Vaccine,* vol. 36, no. 26, pp. 3861-3867, 2018.

[10] J. D. Grabenstein, “What the World's religions teach, applied to vaccines and immune globulins,” *Vaccine,* vol. 31, no. 16, pp. 2011-2023, 2013.

[11] C. H. Gaughan, D. Ayoubkhani, V. Nafilyan, P. Goldblatt, C. White, K. Tingay and N. Bannister, “Religious affiliation and COVID-19-related mortality: A retrospective cohort study of prelockdown and postlockdown risks in England and Wales,” *Journal of Epidemiology and Community Health,* vol. 0, p. 1–6, 1 2021.

[12] D. Ayoubkhani, V. Nafilyan, C. White, P. Goldblatt, C. Gaughan, L. Blackwell, N. Rogers, A. Banerjee, K. Khunti, M. Glickman, B. Humberstone and I. Diamond, “Ethnic-minority groups in England and Wales—factors associated with the size and timing of elevated COVID-19 mortality: a retrospective cohort study linking census and death records,” *International Journal of Epidemiology,* vol. 49, p. 1951–1962, 1 2021.

[13] R. Mathur, C. T. Rentsch, C. E. Morton, W. J. Hulme, A. Schultze, B. MacKenna, R. Eggo, K. Bhaskaran, A. Y. S. Wong, E. J. Williamson, H. Forbes, K. Wing, H. I. McDonald, C. Bates, S. Bacon, A. J. Walker, D. Evans, P. Inglesby, A. Mehrkar, H. J. Curtis, N. J. DeVito, R. Croker, H. Drysdale, J. Cockburn, J. Parry, F. Hester, S. Harper, I. J. Douglas, L. Tomlinson, S. J. W. Evans, R. Grieve, D. Harrison, K. Rowan, K. Khunti, N. Chaturvedi, L. Smeeth and B. Goldacre, “Ethnic differences in COVID-19 infection, hospitalisation, and mortality: an OpenSAFELY analysis of 17 million adults in England,” *medRxiv,* p. 2020.09.22.20198754, 2020.

[14] V. Nafilyan, N. Islam, R. Mathur, D. Ayoubkhani, A. Banerjee, M. Glickman, B. Humberstone, I. Diamond and K. Khunti, “Ethnic differences in COVID-19 mortality during the first two waves of the Coronavirus Pandemic: a nationwide cohort study of 29 million adults in England,” *MedRxiv,* 2021.

[15] S. Palermo, “Covid-19 Pandemic: Maximizing Future Vaccination Treatments Considering Aging and Frailty,” *Frontiers in Medicine,* vol. 7, p. 632, 2020.

For peer review only

Acknowledgements
We are grateful to Charlotte Bermingham for useful discussions about this paper.

Funding
This work was supported by a grant from the UKRI (MRC)-DHSC (NIHR) COVID-19 Rapid Response Rolling Call (MR/V020536/1) and from HDR-UK (HDRUK2020.138). KK, TY and CR are supported by the National Institute for Health Research (NIHR) Applied Research Collaboration East Midlands (ARC EM) and the NIHR Leicester Biomedical Research Centre (BRC). ASW is an NIHR Senior Investigator and is supported by the NIHR Health Protection Research Unit in Healthcare Associated Infections and Antimicrobial Resistance at Oxford University in partnership with Public Health England (PHE) (NIHR200915) and the NIHR Biomedical Research Centre, Oxford. The views expressed in this publication are those of the authors and not necessarily those of the NHS, the National Institute for Health Research, the Department of Health or Public Health England.

Ethical Approval Statement

Ethical approval was obtained from the National Statistician's Data Ethics Advisory Committee (NSDEC(20)12)

Data sharing
Data are not yet available, but will be made available on the ONS Secure Research Service for accredited researchers.

Authorship statement
Study conceptualisation was led by VN, TY and CR. VN, TY, CR, TD, CG, SW, DA, MG, KK contributed to the development of the research question, study design, with development of statistical aspects led by VN and TD. VN, JM and TD were involved in data specification, curation and collection. VN and TD conducted and checked the statistical analyses. VN, TY, CR, TD, CG, JM, SW, DA, MG, KK contributed to the interpretation of the results. VN, TY and CR wrote the first draft of the paper. VN, TY, CR, TD, CG, JM, SW, DA, MG, KK contributed to the critical revision of the manuscript for important intellectual content and approved the final version of the manuscript.
VN had full access to all data in the study and takes responsibility of the integrity of the data and the accuracy of the data analysis. The lead author (VN) affirms that the manuscript is an honest, accurate, and transparent account of the study being reported; that no important aspects of the study have been omitted; and that any discrepancies from the study as planned have been explained

Conflict of interest
KK is Director of the University of Leicester Centre for Black Minority Ethnic Health, Trustee of the South Asian Health Foundation, Chair of the Ethnicity Subgroup of SAGE and Member of Independent SAGE**.**

For peer review only

Tables

Table 1: Variables used in the analyses

Variable	Coding	Source
Vaccinated	Received a first dose of a COVID-19 vaccine by 15th March 2020	NIMS
Age	Third-order polynomial	2011 Census
Sex	Female, Male	2011 Census
Ethnicity	White British, Bangladeshi, Black African, Black Caribbean, Chinese, Indian, Mixed, Other, Pakistani, White other	2011 Census
Religious affiliation	Christian, Buddhist, Hindu, Jewish, Muslim, No religion, Other Religion, Religion Not Stated, Sikh	2011 Census
Region	Dummy variables representing region of residence	2019 NHS PR
Rural urban classification	Urban, rural	2019 NHS PR
Index of Multiple Deprivation (IMD)	Dummy variables representing quintiles of deprivation	2019 NHS PR
Household tenure	Own, social rented, private rented, other	2011 Census
Level of highest qualification	Degree, A-level or equivalent, GCSE or equivalent, no qualification, other	2011 Census
Disability	Non-disabled , disabled (limited a little), disabled (limited a lot)	2011 Census
Body Mass Index (kg/m2)	< 18.5, 18.5 – 25, 25 to 30, >= 30, missing	GPES
Chronic kidney disease (CKD)	No CKD, CKD3, CKD4, CKD5	GPES
Learning disability	No learning disability, Down's Syndrome, other learning disability	GPES
Cancer and immunosuppression	Dummies for blood cancer, solid organ transplant , Prescribed immunosuppressant medication by GP , Prescribed leukotriene or long-acting beta blockers, Prescribed regular prednisolone ,	GPES

For peer review only

Other conditions	Diabetes, Chronic obstructive pulmonary disease (COPD), Asthma , Rare pulmonary diseases , Pulmonary hypertension or pulmonary fibrosis , Coronary heart disease , Stroke , Atrial Fibrillation , Congestive cardiac failure , Venous thromboembolism , Peripheral vascular disease , Congenital heart disease , Dementia , Parkinson's disease , Epilepsy , Rare neurological conditions , Cerebral palsy , Severe mental illness (bipolar disorder, schizophrenia, severe depression), Osteoporotic fracture , Rheumatoid arthritis or Systemic lupus erythematosus , Cirrhosis of the liver	GPES/HES

Table 2: Characteristics of the study population

Variable	Level	Count (%)
Vaccinated		6,202,780 (93.2)
Sex	Female	3,672,314 (55.2)
	Male	2,983,358 (44.8)
Age	Mean (SD)	78.8 (6.5)
Ethnicity	Bangladeshi	11,522 (0.2)
	Black African	21,535 (0.3)
	Black Caribbean	52,883 (0.78)
	Chinese	18,452 (0.38)
	Indian	103,564 (1.6)
	Mixed	24,637 (0.34)
	Other	65,241 (1.0)
	Pakistani	39,723 (0.6)
	White British	6,095,276 (91.6)
	White other	222,839 (3.4)
Religion	Buddhist	16,403 (0.3)
	Christian	5,221,392 (78.5)
	Hindu	61,634 (0.9)
	Jewish	39,800 (0.6)
	Muslim	86,841 (1.3)
	No religion	725,695 (10.9)
	Other Religion	22,327 (0.3)
	Religion Not Stated	449,781 (6.8)
	Sikh	31,799 (0.5)

For peer review only

IMD Quintile	1 (Most deprived)	913,809 (13.7)
	2	1,140,651 (17.1)
	3	1,407,155 (21.1)
	4	1,560,023 (23.4)
	5 (Least deprived)	1,634,034 (24.6)
Household tenure	Owned	5,488,126 (82.5)
	Private rented	273,707 (4.1)
	Social rented	778,867 (11.7)
	Other (eg live rent free)	114,972 (1.7)
Rural-Urban	Rural	6,005,144 (82.4)
	Urban	304,412 (4.2)
Household composition	2 Elderly	847,508 (11.6)
	1 Elderly	128,083 (1.8)
	Care home	436,211 (6.0.)
	Missing household	9,035 (0.1)
	Multigenerational	620,167 (8.5)
	Other (3+ Adults)	714,211 (9.8)

Note: Adults aged 70 years or over, living in England, alive on 15th March 2021.

For peer review only

Table 3: Vaccination rates and odds ratios for not being vaccinated by socio-demographic characteristics

**Exposure**	**Group**	**Vaccination rate**	**Odds ratio (Model 1)**	**Odds ratio (Model 2)**	**Odds ratio (Model 3)**
Age group	70-74	90.9 [90.8 - 90.9]	1 (Ref)	1 (Ref)	1 (Ref)
	75-79	93.8 [93.8 - 93.9]	0.65 [0.65 - 0.66]	0.65 [0.65 - 0.66]	0.66 [0.65 - 0.66]
	80-84	95.6 [95.5 - 95.6]	0.46 [0.46 - 0.46]	0.46 [0.46 - 0.46]	0.45 [0.45 - 0.46]
	85-89	95.1 [95.0 - 95.1]	0.51 [0.51 - 0.52]	0.51 [0.51 - 0.52]	0.51 [0.51 - 0.52]
	90-94	94.0 [93.9 - 94.1]	0.63 [0.62 - 0.64]	0.63 [0.62 - 0.64]	0.64 [0.63 - 0.65]
	95-99	92.3 [92.1 - 92.5]	0.83 [0.81 - 0.85]	0.82 [0.80 - 0.85]	0.82 [0.80 - 0.84]
	100+	85.5 [84.8 - 86.1]	1.69 [1.61 - 1.78]	1.68 [1.60 - 1.77]	1.60 [1.52 - 1.68]
Sex	Female	93.2 [93.2 - 93.2]	1 (Ref)	1 (Ref)	1 (Ref)
	Male	93.2 [93.2 - 93.2]	1.00 [1.00 - 1.01]	0.98 [0.98 - 0.99]	1.03 [1.02 - 1.03]
Disability	Not Limited	93.5 [93.5 - 93.5]	1 (Ref)	1 (Ref)	1 (Ref)
	Limited a little	93.4 [93.3 - 93.4]	1.02 [1.01 - 1.03]	1.12 [1.11 - 1.13]	1.08 [1.07 - 1.08]
	Limited a lot	91.3 [91.2 - 91.3]	1.37 [1.36 - 1.38]	1.47 [1.46 - 1.48]	1.29 [1.28 - 1.30]
Ethnicity	White British	94.0 [94.0 - 94.0]	1 (Ref)	1 (Ref)	1 (Ref)
	Bangladeshi	82.7 [82.0 - 83.4]	3.26 [3.11 - 3.42]	3.48 [3.31 - 3.65]	2.56 [2.43 - 2.69]
	Black African	67.2 [66.6 - 67.8]	7.62 [7.40 - 7.84]	7.59 [7.37 - 7.81]	5.01 [4.86 - 5.16]
	Black Caribbean	73.8 [73.4 - 74.2]	5.55 [5.44 - 5.66]	6.35 [6.22 - 6.47]	4.85 [4.75 - 4.96]
	Chinese	82.8 [82.3 - 83.4]	3.23 [3.11 - 3.36]	3.11 [2.99 - 3.23]	2.64 [2.54 - 2.75]
	Indian	90.9 [90.7 - 91.0]	1.57 [1.54 - 1.60]	1.55 [1.52 - 1.59]	1.35 [1.32 - 1.38]
	Mixed	85.3 [84.9 - 85.7]	2.69 [2.60 - 2.79]	2.67 [2.58 - 2.77]	2.21 [2.14 - 2.30]
	Other	82.9 [82.6 - 83.2]	3.22 [3.15 - 3.29]	3.12 [3.05 - 3.18]	2.44 [2.39 - 2.50]
	Pakistani	79.6 [79.2 - 80.0]	3.99 [3.89 - 4.09]	4.12 [4.02 - 4.22]	3.59 [3.50 - 3.68]
	White other	87.7 [87.5 - 87.8]	2.20 [2.17 - 2.23]	2.24 [2.21 - 2.27]	1.93 [1.90 - 1.95]
IMD Quintile	1 (most deprived)	90.6 [90.6 - 90.7]	1.77 [1.76 - 1.79]	1.76 [1.75 - 1.78]	1.60 [1.59 - 1.62]
	2	92.1 [92.0 - 92.1]	1.48 [1.46 - 1.49]	1.47 [1.46 - 1.48]	1.34 [1.33 - 1.35]
	3	93.4 [93.4 - 93.5]	1.20 [1.19 - 1.22]	1.20 [1.19 - 1.21]	1.17 [1.16 - 1.18]
	4	94.0 [94.0 - 94.0]	1.09 [1.08 - 1.10]	1.09 [1.08 - 1.10]	1.09 [1.08 - 1.10]
	5 (least deprived)	94.5 [94.4 - 94.5]	1 (Ref)	1 (Ref)	1 (Ref)
Religion	Christian	93.8 [93.8 - 93.9]	1 (Ref)	1 (Ref)	1 (Ref)
	Buddhist	84.1 [83.5 - 84.7]	2.88 [2.76 - 3.01]	2.63 [2.52 - 2.74]	2.03 [1.95 - 2.12]
	Hindu	91.5 [91.2 - 91.7]	1.43 [1.39 - 1.47]	1.39 [1.35 - 1.43]	1.03 [1.00 - 1.06]
	Jewish	93.1 [92.8 - 93.3]	1.13 [1.09 - 1.18]	1.12 [1.08 - 1.17]	0.94 [0.90 - 0.97]
	Muslim	79.1 [78.9 - 79.4]	4.02 [3.95 - 4.09]	4.04 [3.97 - 4.11]	2.74 [2.69 - 2.79]
	No religion	91.9 [91.9 - 92.0]	1.34 [1.33 - 1.35]	1.25 [1.23 - 1.26]	1.23 [1.22 - 1.24]
	Other Religion	85.4 [84.9 - 85.8]	2.61 [2.51 - 2.71]	2.41 [2.32 - 2.50]	2.15 [2.07 - 2.23]
	Religion Not Stated	91.5 [91.4 - 91.6]	1.42 [1.41 - 1.44]	1.40 [1.38 - 1.41]	1.35 [1.33 - 1.36]
	Sikh	91.6 [91.3 - 91.9]	1.39 [1.34 - 1.45]	1.35 [1.30 - 1.41]	1.07 [1.03 - 1.11]
Household tenure	Owned	94.0 [93.9 - 94.0]	1 (Ref)	1 (Ref)	1 (Ref)
	Other	91.1 [90.9 - 91.2]	1.52 [1.49 - 1.55]	1.55 [1.52 - 1.58]	1.49 [1.46 - 1.52]

	Private rented	88.4 [88.2 - 88.5]	2.05 [2.02 - 2.07]	1.96 [1.94 - 1.99]	1.81 [1.79 - 1.83]
	Social rented	89.9 [89.8 - 89.9]	1.75 [1.73 - 1.76]	1.77 [1.76 - 1.79]	1.60 [1.59 - 1.61]
Rural-Urban	Rural	94.5 [94.4 - 94.5]	1 (Ref)	1 (Ref)	1 (Ref)
	Urban	92.8 [92.8 - 92.8]	1.33 [1.32 - 1.34]	1.34 [1.33 - 1.35]	1.12 [1.12 - 1.13]
Household composition	2 Elderly	94.5 [94.4 - 94.5]	1 (Ref)	1 (Ref)	1 (Ref)
	1 Elderly	92.5 [92.5 - 92.6]	1.38 [1.37 - 1.39]	1.53 [1.52 - 1.54]	1.32 [1.31 - 1.33]
	Care home	94.9 [94.8 - 95.0]	0.92 [0.89 - 0.95]	1.10 [1.06 - 1.13]	0.89 [0.86 - 0.91]
	Multigenerational	90.3 [90.3 - 90.4]	1.83 [1.82 - 1.85]	1.77 [1.76 - 1.79]	1.39 [1.38 - 1.40]
	Other (3+ Adults)	90.0 [89.6 - 90.3]	1.91 [1.84 - 1.97]	1.81 [1.75 - 1.88]	1.54 [1.49 - 1.60]

Note: Adults aged 70 years or over, living in England, alive on 15th March 2021. Model 1: unadjusted; Model 2: adjusted for sex and age (Cubic splines); Model 3: adjusted for sex, age (Cubic splines), care home status, rural/urban, region, ethnicity (except when looking at religion as an exposure), IMD quintile (except when looking at household tenure as an exposure), disability, BMI and comorbidities. See Table 1 for more details on the variables included in the models.

For peer review only

STROBE Statement—Checklist of items that should be included in reports of ***cohort studies***

	Item No	Recommendation	Page No
**Title and abstract**	1	(*a*) Indicate the study's design with a commonly used term in the title or the abstract	1
		(*b*) Provide in the abstract an informative and balanced summary of what was done and what was found	2
**Introduction**			
Background/rationale	2	Explain the scientific background and rationale for the investigation being reported	3
Objectives	3	State specific objectives, including any prespecified hypotheses	3
**Methods**			
Study design	4	Present key elements of study design early in the paper	3
Setting	5	Describe the setting, locations, and relevant dates, including periods of recruitment, exposure, follow-up, and data collection	3
Participants	6	(*a*) Give the eligibility criteria, and the sources and methods of selection of participants. Describe methods of follow-up	3
		(*b*) For matched studies, give matching criteria and number of exposed and unexposed	
Variables	7	Clearly define all outcomes, exposures, predictors, potential confounders, and effect modifiers. Give diagnostic criteria, if applicable	3
Data sources/ measurement	8*	For each variable of interest, give sources of data and details of methods of assessment (measurement). Describe comparability of assessment methods if there is more than one group	3
Bias	9	Describe any efforts to address potential sources of bias	3
Study size	10	Explain how the study size was arrived at	3
Quantitative variables	11	Explain how quantitative variables were handled in the analyses. If applicable, describe which groupings were chosen and why	4 Table S1
Statistical methods	12	(*a*) Describe all statistical methods, including those used to control for confounding	4
		(*b*) Describe any methods used to examine subgroups and interactions	NA
		(*c*) Explain how missing data were addressed	NA
		(*d*) If applicable, explain how loss to follow-up was addressed	NA
		(*e*) Describe any sensitivity analyses	NA
**Results**			
Participants	13*	(a) Report numbers of individuals at each stage of study—eg numbers potentially eligible, examined for eligibility, confirmed eligible, included in the study, completing follow-up, and analysed	3,4
		(b) Give reasons for non-participation at each stage	3
		(c) Consider use of a flow diagram	
Descriptive data	14*	(a) Give characteristics of study participants (eg demographic, clinical, social) and information on exposures and potential confounders	4 (Table1)
		(b) Indicate number of participants with missing data for each variable of interest	4 (Table 1)
		(c) Summarise follow-up time (eg, average and total amount)	
Outcome data	15*	Report numbers of outcome events or summary measures over time	Table 1

For peer review only

Main results	16	(*a*) Give unadjusted estimates and, if applicable, confounder-adjusted estimates and their precision (eg, 95% confidence interval). Make clear which confounders were adjusted for and why they were included<br>(*b*) Report category boundaries when continuous variables were categorized<br>(*c*) If relevant, consider translating estimates of relative risk into absolute risk for a meaningful time period	4
Other analyses	17	Report other analyses done—eg analyses of subgroups and interactions, and sensitivity analyses	
**Discussion**			
Key results	18	Summarise key results with reference to study objectives	5
Limitations	19	Discuss limitations of the study, taking into account sources of potential bias or imprecision. Discuss both direction and magnitude of any potential bias	5
Interpretation	20	Give a cautious overall interpretation of results considering objectives, limitations, multiplicity of analyses, results from similar studies, and other relevant evidence	5
Generalisability	21	Discuss the generalisability (external validity) of the study results	5
**Other information**			
Funding	22	Give the source of funding and the role of the funders for the present study and, if applicable, for the original study on which the present article is based	7

*Give information separately for exposed and unexposed groups.

Note: An Explanation and Elaboration article discusses each checklist item and gives methodological background and published examples of transparent reporting. The STROBE checklist is best used in conjunction with this article (freely available on the Web sites of PLoS Medicine at http://www.plosmedicine.org/, Annals of Internal Medicine at http://www.annals.org/, and Epidemiology at http://www.epidem.com/). Information on the STROBE Initiative is available at http://www.strobe-statement.org.

For peer review only

BMJ Open

Sociodemographic inequality in COVID-19 vaccination coverage amongst elderly adults in England: a national linked data study

Journal:	*BMJ Open*
Manuscript ID	bmjopen-2021-053402.R2
Article Type:	Original research
Date Submitted by the Author:	08-Jul-2021
Complete List of Authors:	Nafilyan, Vahe; Office for National Statistics, ; London School of Hygiene & Tropical Medicine,<br>Dolby, Ted; Office for National Statistics<br>Razieh, Cameron; University of Leicester; University Hospitals of Leicester NHS Trust<br>Gaughan, Charlotte; Office for National Statistics, Methodology<br>Morgan, Jasper; Office for National Statistics<br>Ayoubkhani, Daniel; Office for National Statistics, Methodology<br>Walker, Sarah; University of Oxford, Nuffield department of Medicine<br>Khunti, Kamlesh; University of Leicester; Leicester General Hospital<br>Glickman, Myer; Office for National Statistics<br>Yates, Thomas; University of Leicester, Diabetes Research Centre
**Primary Subject Heading**:	Public health
Secondary Subject Heading:	Health policy
Keywords:	COVID-19, Infection control < INFECTIOUS DISEASES, Epidemiology < INFECTIOUS DISEASES

SCHOLARONE™
Manuscripts

I, the Submitting Author has the right to grant and does grant on behalf of all authors of the Work (as defined in the below author licence), an exclusive licence and/or a non-exclusive licence for contributions from authors who are: i) UK Crown employees; ii) where BMJ has agreed a CC-BY licence shall apply, and/or iii) in accordance with the terms applicable for US Federal Government officers or employees acting as part of their official duties; on a worldwide, perpetual, irrevocable, royalty-free basis to BMJ Publishing Group Ltd ("BMJ") its licensees and where the relevant Journal is co-owned by BMJ to the co-owners of the Journal, to publish the Work in this journal and any other BMJ products and to exploit all rights, as set out in our licence.

The Submitting Author accepts and understands that any supply made under these terms is made by BMJ to the Submitting Author unless you are acting as an employee on behalf of your employer or a postgraduate student of an affiliated institution which is paying any applicable article publishing charge ("APC") for Open Access articles. Where the Submitting Author wishes to make the Work available on an Open Access basis (and intends to pay the relevant APC), the terms of reuse of such Open Access shall be governed by a Creative Commons licence – details of these licences and which Creative Commons licence will apply to this Work are set out in our licence referred to above.

Other than as permitted in any relevant BMJ Author's Self Archiving Policies, I confirm this Work has not been accepted for publication elsewhere, is not being considered for publication elsewhere and does not duplicate material already published. I confirm all authors consent to publication of this Work and authorise the granting of this licence.

For peer review only

Sociodemographic inequality in COVID-19 vaccination coverage amongst elderly adults in England: a national linked data study

Vahé Nafilyan[1,2], Ted Dolby[2], Cameron Razieh[3,4] , Charlotte Gaughan[2], Jasper Morgan[2], Daniel Ayoubkhani[2], A. Sarah Walker[5], Kamlesh Khunti[3,4], Myer Glickman[2], Thomas Yates[3,4]

[1] Faculty of Public Health, Environment and Society, London School of Hygiene and Tropical Medicine, London, UK

[2] Office for National Statistics, Newport, UK

[3] Diabetes Research Centre, University of Leicester, Leicester, UK

[4] NIHR Leicester Biomedical Research Centre, University of Leicester and University Hospitals of Leicester NHS Trust, Leicester, UK

[5]Nuffield department of Medicine, University of Oxford, Oxford, UK

Corresponding author:

Vahé Nafilyan (vahe.nafilyan@ons.gov.uk)

For peer review only

Abstract

Objective: To examine inequalities in COVID-19 vaccination rates amongst elderly adults in England

Design: Cohort study

Setting: People living in private households and communal establishments in England

Participants: 6,829,643 adults aged ≥70 years (mean 78.7 years, 55.2% female) who were alive on 15 March 2021.

Main outcome measures: Having received the first dose of a vaccine against COVID-19 by 15 March 2021. We calculated vaccination rates and estimated unadjusted and adjusted odds ratios using logistic regression models.

Results: By 15 March 2021, 93.2% of people living in England aged 70 years and over had received at least one dose of a COVID-19 vaccine. While vaccination rates differed across all factors considered apart from sex, the greatest disparities were seen between ethnic and religious groups. The lowest rates were in people of Black African and Black Caribbean ethnic backgrounds, where only 67.2% and 73.9% had received a vaccine, with adjusted odds of not being vaccinated at 5.01 (95% CI 4.86 - 5.16) and 4.85 (4.75 - 4.96) times greater than the White British group. The proportion of individuals self-identifying as Muslim and Buddhist who had received a vaccine was 79.1% and 84.1%, respectively. Older age, greater area deprivation, less advantaged socio-economic position (proxied by living in a rented home), being disabled and living either alone or in a multi-generational household were also associated with higher odds of not having received the vaccine.

Conclusion: Research is now urgently needed to understand why disparities exist in these groups and how they can best be addressed through public health policy and community engagement.

For peer review only

Summary Box

Strengths and limitations of this study

- The main strength of our population-level dataset is the availability of a wide range of socio-demographic characteristics not included in electronic health records, allowing for a detailed examination of inequalities in vaccination coverage.
- We presented vaccination rates and odds ratios for non-vaccination adjusted for a range of factors to understand further inequalities in vaccination coverage.
- The main limitation is that most demographic and socio-economic characteristics were derived from the 2011 Census and therefore are ten years old
- Because the dataset is based on the 2011 Census, it excluded people living in England in 2011 but not taking part in the 2011 Census, respondents who could not be linked to the 2011-2013 NHS patients register and recent migrants.

For peer review only

Introduction

The UK began an ambitious vaccination programme to combat the COVID-19 pandemic on 8th December 2020; by 24th April 2021, 64% of the UK adult population have received their first of the dose [1].

Previous research demonstrates that vaccination rates tend to be lower amongst certain ethnic groups, and in areas of higher deprivation [2, 3, 4]. Existing evidence suggests that COVID-19 vaccination rates differ by level of area deprivation, certain underlying health conditions and ethnicity [5]. Far less is known about how COVID-19 vaccination uptake varies by socio-demographic factors, such as religious affiliation, individual socio-economic status, living in multigenerational household or disability status, factors disproportionately associated with SARS-CoV-2 infection. Understanding which socio-demographic, economic, and cultural factors are associated with low vaccination rates has major implications for designing policies that help maximise the vaccination campaign coverage.

This study investigates inequality in vaccination rates amongst adults aged ≥70 years in England, using population-level administrative records linked to the 2011 Census. This enables examination of a wide range of socio-demographic characteristics, currently lacking in previously published studies, in particular ethnicity, religion, different measures of socio-economic position, and those who report being disabled.

Methods

Study data

We linked vaccination data from the National Immunisation Management System (NIMS) to the Office for National Statistics (ONS) Public Health Data Asset (PHDA) based on NHS number. The ONS PHDA is a linked dataset combining the 2011 Census, mortality records, the General Practice Extraction Service (GPES) data for pandemic planning and research and the Hospital Episode Statistics (HES). To obtain NHS numbers for the 2011 Census, we linked the 2011 Census to the 2011-2013 NHS Patient Registers using deterministic and probabilistic matching, with an overall linkage rate of 94.6%. All subsequent linkages were performed based on NHS numbers.

The study population consisted of people aged ≥70 years, alive on 15th March 2020, who were resident in England, registered with a general practitioner, and enumerated at the 2011 Census. Of 6,605,315 adults aged ≥70 years who received a first dose of a COVID-19 vaccine in NIMS, 6,242,384 (94.5%) were linked to the ONS PHDA.

Outcome

The main outcome was having received at least a first dose of a COVID-19 vaccine by 15th March 2021, as recorded in the NIMS data available on 31st March 2021. Phase 1 of the vaccination policy for England aimed to offer a first vaccination appointment to all those ≥70 years by 15th February, and we allowed a further month to ensure full coverage.

Exposures and covariates

This dataset combines comprehensive socio-demographic information from the 2011 Census with a detailed medical history from clinical records. All individual-level socio-demographic characteristics

For peer review only - http://bmjopen.bmj.com/site/about/guidelines.xhtml

(ethnic group, religious affiliation, disability status, educational attainment) came from the 2011 Census. We used a 10-category ethnic group classification (white British, Bangladeshi, black African, black Caribbean, Chinese, Indian, Mixed, Other, Pakistani, white other). Self-reported religious group, place of residence (region within England, private or care home) and area-based deprivation (Index of Multiple Deprivation [6]) were derived based on the 2019 Patient Register. Comorbidities were defined as in the QCOVID risk prediction model, a model used to assess the risk of severe COVID-19 outcomes in the general population, used to inform the prioritisation of the vaccination campaign [7]. All variables included in this analysis are listed in Table 1.

[Table 1]

Statistical analyses

First, we estimated the first dose vaccination rates by a range of demographic and socio-economic characteristics. Second, to understand the drivers of the observed differences in vaccination rates, we used logistic regression to estimate the odds of not having received a first dose of a COVID-19 vaccine. For each exposure, we compared odds ratios (ORs) from models adjusted for different sets of covariates. We estimated unadjusted ORs, ORs adjusted for sex and age, and ORs adjusted for all geographic and socio-demographic characteristics, disability status and pre-existing conditions. All analyses were conducted using R 3.5

Patient and Public Involvement

No patient involved

Results

Our study population included 6,724,179 adults aged ≥70 years who lived in England. 55.1% were women and the mean age was 78.8 (SD: 6.5) years; 91.6% identified themselves as white British, 78.5% as Christian. 82.5% owned their home (Table 2). By 15 March 2021, 93.2% of people living in England aged 70 years and over had received at least one dose of a COVID-19 vaccine.

[Table 2]

Table 3 shows vaccination rates by demographic and socio-economic factors, as well as odds ratios (OR) from different models. Vaccination rates differed across all factors considered, apart from sex. The lowest rates were in people of Black African and Black Caribbean ethnic backgrounds where only 67.2% and 73.9% had received a vaccine. Adjusting for differences in geography, socio-demographic factors and underlying health conditions did not fully explain the lower probability of having received the vaccine among ethnic minority groups. Compared with people of white British ethnicity, the fully adjusted OR for black African individuals was 5.01 [95% CI 4.86 - 5.16], whilst the unadjusted OR was 7.62 [7.40 - 7.84], suggesting that geography, socio-demographic factors and pre-pandemic health only explains about 40% of the elevated odds of not being vaccinated.

Vaccination rates also varied markedly across religious groups. Whilst 93.8% of Christians had been vaccinated, only 79.1% of Muslims and 84.1% of Buddhists had been vaccinated. Stark differences remained after adjustment for other factors, with an adjusted OR of not being vaccinated of 2.74 [2.69 - 2.79] for Muslims and 2.03 [1.95 - 2.12] for Buddhist, compared to Christians.

For peer review only - http://bmjopen.bmj.com/site/about/guidelines.xhtml

For peer review only

Greater area deprivation, less advantaged socio-economic position (proxied by living in a rented home), being disabled and living either alone or in a multi-generational household were also associated with low vaccination rates, even when adjusting for other factors (Table 3). These differences were less pronounced than the differences between ethnic groups or religious affiliations.

[Table 3]

Discussion

Main findings

Our analysis using whole population level linked data in England suggests that first dose vaccination rates in adults aged ≥70 differed markedly by ethnic group and self-reported religious affiliation. The percentage of people vaccinated was lower among all minority ethnic groups compared to the White British population, with the lowest vaccination rates observed among Black African, Black Caribbean, Bangladeshi and Pakistani individuals. In addition, lower vaccination rates were reported among individuals who identified as Muslim and Buddhist. Whilst some differences were found by deprivation, household factors, disability status and other socio-demographic factors, these were less pronounced compared with ethnicity or religious affiliation.

Comparison with other studies

Few studies have investigated how COVID-19 vaccination coverage varies by a wide range of socio-demographic characteristics. Our results on ethnicity and area deprivation are consistent with one previous study based on clinical records for 40% of patients in England [3]. In addition, our results confirm studies showing that influenza, shingles and pneumococcal vaccination are patterned by similar factors, including ethnicity, deprivation and household size [8]. Pre-pandemic, religion and culture have been postulated to be important factors in determining vaccination uptake [9]; our results extend this by showing that self-reported religious affiliation is an important factor in COVID-19 vaccine uptake. Differences in vaccination rate and potential vaccination hesitancy between religious groups may not be based upon religious beliefs, but rather reflect safety and other concerns [10], or, given high infection rates in some of these groups [11], beliefs that vaccination is not needed after natural infection. We also find that vaccination rates vary by individual characteristics not reported in previous studies, such as household tenure (a proxy for socio-economic status), household composition and disability status.

Strengths and limitation

The primary study strength is using nationwide linked population-level data from clinical records and the 2011 Census. Unlike studies based solely on electronic health records, we examined a wide range of socio-demographic characteristics. Unlike surveys, we can precisely estimate vaccination rates and odds ratios for small groups. The main limitation is that most demographic and socio-economic characteristics are derived from the 2011 Census and therefore are 10 years old. However, we focus primarily on characteristics that are unlikely to change over time, such as ethnicity or religion, or likely to be stable for our population (adults aged ≥70), such as household tenure. However, for the characteristics likely to change over time, such as disability status, the time difference may introduce some bias into the

For peer review only

estimates, although this would be expected to dilute differences, since we are most likely missing some long-term health conditions. Care home residency and area deprivation were derived from the 2019 Patient register and are therefore not subject to the same biases. Another limitation is that because the Public Health Data Asset was based on the 2011 Census, it excluded people living in England in 2011 but not taking part in the 2011 Census; respondents who could not be linked to the 2011-2013 NHS patients register; recent migrants. Consequently, we excluded 5.4% of vaccinated people who could not be linked to the ONS PHDA.

Conclusion

There are stark differences in COVID-19 vaccination rates by ethnic group and religious affiliation. Research is now urgently needed to understand why these disparities exist in these groups and how they can best be addressed through public health policy and community engagement. Understanding barriers and supporting participation in the vaccine programme is especially important because the groups with low vaccination coverage were also at elevated risk of COVID-19 mortality in the first two waves of the pandemic [12, 11, 13, 14], are associated with factors, such as frailty, that will continue to elevate risk as the pandemic evolves [15]

References

[1] UK Coronavirus Dashboard, “Coronavirus (COVID-19) in the UK - Vaccinations,” [Online]. Available: https://coronavirus.data.gov.uk/details/vaccinations. [Accessed 27 04 2021].

[2] R. G. Pebody, J. Hippisley-Cox, S. Harcourt, M. Pringle, M. Painter and G. Smith, “Uptake of pneumococcal polysaccharide vaccine in at-risk populations in England and Wales 1999-2005,” *Epidemiology and Infection,* vol. 136, p. 360–369, 3 2008.

[3] A. M. Spencer, S. A. Roberts, L. Brabin, J. Patnick and A. Verma, “Sociodemographic factors predicting mother's cervical screening and daughter's HPV vaccination uptake,” *Journal of Epidemiology and Community Health,* vol. 68, p. 571–577, 2014.

[4] D. Hungerford, P. Macpherson, S. Farmer, S. Ghebrehewet, D. Seddon, R. Vivancos and A. Keenan, “Effect of socioeconomic deprivation on uptake of measles, mumps and rubella vaccination in Liverpool, UK over 16 years: A longitudinal ecological study,” *Epidemiology and Infection,* vol. 144, p. 1201–1211, 4 2016.

[5] B. MacKenna, H. J. Curtis, C. E. Morton, P. Inglesby, A. J. Walker, J. Morley, A. Mehrkar, S. Bacon, G. Hickman, C. Bates, R. Croker, D. Evans, T. Ward, J. Cockburn, S. Davy, K. Bhaskaran, A. Schultze, C. T. Rentsch, E. Williamson, W. Hulme, H. I. McDonald, L. Tomlinson, R. Mathur, H. Drysdale, R. M. Eggo, K. Wing, A. Y. S. Wong, H. Forbes, J. Parry, F. Hester, S. Harper, I. J. Douglas, S. J. W. Evans, L. Smeeth and B. Goldacre, “Trends, regional variation, and clinical characteristics of COVID-19 vaccine recipients: a retrospective cohort study in 23.4 million patients using OpenSAFELY,” *medRxiv,* 2021.

For peer review only

[6] Department for Communities and Local Government, “The English Indices of Deprivation 2015,” 2015. [Online]. Available: https://www.gov.uk/government/statistics/english-indices-of-deprivation-2015. [Accessed 3 May 2021].

[7] A. K. Clift, C. A. C. Coupland, R. H. Keogh, K. Diaz-Ordaz, E. Williamson, E. M. Harrison, A. Hayward, H. Hemingway, P. Horby, N. Mehta, J. Benger, K. Khunti, D. Spiegelhalter, A. Sheikh, J. Valabhji, R. A. Lyons, J. Robson, M. G. Semple, F. Kee, P. Johnson, S. Jebb, T. Williams and J. Hippisley-Cox, “Living risk prediction algorithm (QCOVID) for risk of hospital admission and mortality from coronavirus 19 in adults: national derivation and validation cohort study,” *BMJ,* vol. 371, 2020.

[8] P. S. Tan, M. Patone, A. K. Clift, H. Dambha-Miller, D. Saatci, T. Ranger, C. Garriga, F. Zaccardi, B. R. Shah, C. Coupland, S. Griffin, K. Khunti and J. Hippisley-Cox, “Influenza, Shingles and Pneumococcal Vaccine Uptake, Offer and Refusal in Adult Populations at High-Risk for COVID-19: A UK Population-Based Cohort Study,” *SSRN Electronic Journal,* 2021.

[9] S. Lane, N. E. MacDonald, M. Marti and L. Dumolard, “Vaccine hesitancy around the globe: Analysis of three years of WHO/UNICEF Joint Reporting Form data-2015–2017,” *Vaccine,* vol. 36, no. 26, pp. 3861-3867, 2018.

[10] J. D. Grabenstein, “What the World's religions teach, applied to vaccines and immune globulins,” *Vaccine,* vol. 31, no. 16, pp. 2011-2023, 2013.

[11] C. H. Gaughan, D. Ayoubkhani, V. Nafilyan, P. Goldblatt, C. White, K. Tingay and N. Bannister, “Religious affiliation and COVID-19-related mortality: A retrospective cohort study of prelockdown and postlockdown risks in England and Wales,” *Journal of Epidemiology and Community Health,* vol. 0, p. 1–6, 1 2021.

[12] D. Ayoubkhani, V. Nafilyan, C. White, P. Goldblatt, C. Gaughan, L. Blackwell, N. Rogers, A. Banerjee, K. Khunti, M. Glickman, B. Humberstone and I. Diamond, “Ethnic-minority groups in England and Wales—factors associated with the size and timing of elevated COVID-19 mortality: a retrospective cohort study linking census and death records,” *International Journal of Epidemiology,* vol. 49, p. 1951–1962, 1 2021.

[13] R. Mathur, C. T. Rentsch, C. E. Morton, W. J. Hulme, A. Schultze, B. MacKenna, R. Eggo, K. Bhaskaran, A. Y. S. Wong, E. J. Williamson, H. Forbes, K. Wing, H. I. McDonald, C. Bates, S. Bacon, A. J. Walker, D. Evans, P. Inglesby, A. Mehrkar, H. J. Curtis, N. J. DeVito, R. Croker, H. Drysdale, J. Cockburn, J. Parry, F. Hester, S. Harper, I. J. Douglas, L. Tomlinson, S. J. W. Evans, R. Grieve, D. Harrison, K. Rowan, K. Khunti, N. Chaturvedi, L. Smeeth and B. Goldacre, “Ethnic differences in COVID-19 infection, hospitalisation, and mortality: an OpenSAFELY analysis of 17 million adults in England,” *medRxiv,* p. 2020.09.22.20198754, 2020.

[14] V. Nafilyan, N. Islam, R. Mathur, D. Ayoubkhani, A. Banerjee, M. Glickman, B. Humberstone, I. Diamond and K. Khunti, “Ethnic differences in COVID-19 mortality during the first two waves of the Coronavirus Pandemic: a nationwide cohort study of 29 million adults in England,” *MedRxiv,* 2021.

[15] S. Palermo, “Covid-19 Pandemic: Maximizing Future Vaccination Treatments Considering Aging and Frailty,” *Frontiers in Medicine,* vol. 7, p. 632, 2020.

For peer review only

Acknowledgements

We are grateful to Charlotte Bermingham for useful discussions about this paper.

Funding

This work was supported by a grant from the UKRI (MRC)-DHSC (NIHR) COVID-19 Rapid Response Rolling Call (MR/V020536/1) and from HDR-UK (HDRUK2020.138). KK, TY and CR are supported by the National Institute for Health Research (NIHR) Applied Research Collaboration East Midlands (ARC EM) and the NIHR Leicester Biomedical Research Centre (BRC). ASW is an NIHR Senior Investigator and is supported by the NIHR Health Protection Research Unit in Healthcare Associated Infections and Antimicrobial Resistance at Oxford University in partnership with Public Health England (PHE) (NIHR200915) and the NIHR Biomedical Research Centre, Oxford. The views expressed in this publication are those of the authors and not necessarily those of the NHS, the National Institute for Health Research, the Department of Health or Public Health England.

Ethical Approval Statement

Ethical approval was obtained from the National Statistician's Data Ethics Advisory Committee (NSDEC(20)12)

Data sharing

Data are not yet available, but will be made available on the ONS Secure Research Service for accredited researchers.

Authorship statement

Study conceptualisation was led by VN, TY and CR. VN, TY, CR, TD, CG, SW, DA, MG, KK contributed to the development of the research question, study design, with development of statistical aspects led by VN and TD. VN, JM and TD were involved in data specification, curation and collection. VN and TD conducted and checked the statistical analyses. VN, TY, CR, TD, CG, JM, SW, DA, MG, KK contributed to the interpretation of the results. VN, TY and CR wrote the first draft of the paper. VN, TY, CR, TD, CG, JM, SW, DA, MG, KK contributed to the critical revision of the manuscript for important intellectual content and approved the final version of the manuscript.

VN had full access to all data in the study and takes responsibility of the integrity of the data and the accuracy of the data analysis. The lead author (VN) affirms that the manuscript is an honest, accurate, and transparent account of the study being reported; that no important aspects of the study have been omitted; and that any discrepancies from the study as planned have been explained

Conflict of interest

KK is Director of the University of Leicester Centre for Black Minority Ethnic Health, Trustee of the South Asian Health Foundation, Chair of the Ethnicity Subgroup of SAGE and Member of Independent SAGE.

For peer review only

Tables

Table 1: Variables used in the analyses

Variable	Coding	Source
Vaccinated	Received a first dose of a COVID-19 vaccine by 15th March 2020	NIMS
Age	Third-order polynomial	2011 Census
Sex	Female, Male	2011 Census
Ethnicity	White British, Bangladeshi, Black African, Black Caribbean, Chinese, Indian, Mixed, Other, Pakistani, White other	2011 Census
Religious affiliation	Christian, Buddhist, Hindu, Jewish, Muslim, No religion, Other Religion, Religion Not Stated, Sikh	2011 Census
Region	Dummy variables representing region of residence	2019 NHS PR
Rural urban classification	Urban, rural	2019 NHS PR
Index of Multiple Deprivation (IMD)	Dummy variables representing quintiles of deprivation	2019 NHS PR
Household tenure	Own, social rented, private rented, other	2011 Census
Level of highest qualification	Degree, A-level or equivalent, GCSE or equivalent, no qualification, other	2011 Census
Disability	Non-disabled , disabled (limited a little), disabled (limited a lot)	2011 Census
Body Mass Index (kg/m2)	< 18.5, 18.5 – 25, 25 to 30, >= 30, missing	GPES
Chronic kidney disease (CKD)	No CKD, CKD3, CKD4, CKD5	GPES
Learning disability	No learning disability, Down's Syndrome, other learning disability	GPES
Cancer and immunosuppression	Dummies for blood cancer, solid organ transplant , Prescribed immunosuppressant medication by GP , Prescribed leukotriene or long-acting beta blockers, Prescribed regular prednisolone ,	GPES

For peer review only

Other conditions	Diabetes, Chronic obstructive pulmonary disease (COPD), Asthma , Rare pulmonary diseases , Pulmonary hypertension or pulmonary fibrosis , Coronary heart disease , Stroke , Atrial Fibrillation , Congestive cardiac failure , Venous thromboembolism , Peripheral vascular disease , Congenital heart disease , Dementia , Parkinson's disease , Epilepsy , Rare neurological conditions , Cerebral palsy , Severe mental illness (bipolar disorder, schizophrenia, severe depression), Osteoporotic fracture , Rheumatoid arthritis or Systemic lupus erythematosus , Cirrhosis of the liver	GPES/HES

Table 2: Characteristics of the study population

Variable	Level	Count (%)
Vaccinated		6,202,780 (93.2)
Sex	Female	3,672,314 (55.2)
	Male	2,983,358 (44.8)
Age	Mean (SD)	78.8 (6.5)
Ethnicity	Bangladeshi	11,522 (0.2)
	Black African	21,535 (0.3)
	Black Caribbean	52,883 (0.78)
	Chinese	18,452 (0.38)
	Indian	103,564 (1.6)
	Mixed	24,637 (0.34)
	Other	65,241 (1.0)
	Pakistani	39,723 (0.6)
	White British	6,095,276 (91.6)
	White other	222,839 (3.4)
Religion	Buddhist	16,403 (0.3)
	Christian	5,221,392 (78.5)
	Hindu	61,634 (0.9)
	Jewish	39,800 (0.6)
	Muslim	86,841 (1.3)
	No religion	725,695 (10.9)
	Other Religion	22,327 (0.3)
	Religion Not Stated	449,781 (6.8)
	Sikh	31,799 (0.5)

For peer review only

IMD Quintile	1 (Most deprived)	913,809 (13.7)
	2	1,140,651 (17.1)
	3	1,407,155 (21.1)
	4	1,560,023 (23.4)
	5 (Least deprived)	1,634,034 (24.6)
Household tenure	Owned	5,488,126 (82.5)
	Private rented	273,707 (4.1)
	Social rented	778,867 (11.7)
	Other (eg live rent free)	114,972 (1.7)
Rural-Urban	Rural	6,005,144 (82.4)
	Urban	304,412 (4.2)
Household composition	2 Elderly	847,508 (11.6)
	1 Elderly	128,083 (1.8)
	Care home	436,211 (6.0.)
	Missing household	9,035 (0.1)
	Multigenerational	620,167 (8.5)
	Other (3+ Adults)	714,211 (9.8)

Note: Adults aged 70 years or over, living in England, alive on 15th March 2021.

For peer review only

Table 3: Vaccination rates and odds ratios for not being vaccinated by socio-demographic characteristics

**Exposure**	**Group**	**Vaccination rate**	**Odds ratio (Model 1)**	**Odds ratio (Model 2)**	**Odds ratio (Model 3)**
Age group	70-74	90.9 [90.8 - 90.9]	1 (Ref)	1 (Ref)	1 (Ref)
	75-79	93.8 [93.8 - 93.9]	0.65 [0.65 - 0.66]	0.65 [0.65 - 0.66]	0.66 [0.65 - 0.66]
	80-84	95.6 [95.5 - 95.6]	0.46 [0.46 - 0.46]	0.46 [0.46 - 0.46]	0.45 [0.45 - 0.46]
	85-89	95.1 [95.0 - 95.1]	0.51 [0.51 - 0.52]	0.51 [0.51 - 0.52]	0.51 [0.51 - 0.52]
	90-94	94.0 [93.9 - 94.1]	0.63 [0.62 - 0.64]	0.63 [0.62 - 0.64]	0.64 [0.63 - 0.65]
	95-99	92.3 [92.1 - 92.5]	0.83 [0.81 - 0.85]	0.82 [0.80 - 0.85]	0.82 [0.80 - 0.84]
	100+	85.5 [84.8 - 86.1]	1.69 [1.61 - 1.78]	1.68 [1.60 - 1.77]	1.60 [1.52 - 1.68]
Sex	Female	93.2 [93.2 - 93.2]	1 (Ref)	1 (Ref)	1 (Ref)
	Male	93.2 [93.2 - 93.2]	1.00 [1.00 - 1.01]	0.98 [0.98 - 0.99]	1.03 [1.02 - 1.03]
Disability	Not Limited	93.5 [93.5 - 93.5]	1 (Ref)	1 (Ref)	1 (Ref)
	Limited a little	93.4 [93.3 - 93.4]	1.02 [1.01 - 1.03]	1.12 [1.11 - 1.13]	1.08 [1.07 - 1.08]
	Limited a lot	91.3 [91.2 - 91.3]	1.37 [1.36 - 1.38]	1.47 [1.46 - 1.48]	1.29 [1.28 - 1.30]
Ethnicity	White British	94.0 [94.0 - 94.0]	1 (Ref)	1 (Ref)	1 (Ref)
	Bangladeshi	82.7 [82.0 - 83.4]	3.26 [3.11 - 3.42]	3.48 [3.31 - 3.65]	2.56 [2.43 - 2.69]
	Black African	67.2 [66.6 - 67.8]	7.62 [7.40 - 7.84]	7.59 [7.37 - 7.81]	5.01 [4.86 - 5.16]
	Black Caribbean	73.8 [73.4 - 74.2]	5.55 [5.44 - 5.66]	6.35 [6.22 - 6.47]	4.85 [4.75 - 4.96]
	Chinese	82.8 [82.3 - 83.4]	3.23 [3.11 - 3.36]	3.11 [2.99 - 3.23]	2.64 [2.54 - 2.75]
	Indian	90.9 [90.7 - 91.0]	1.57 [1.54 - 1.60]	1.55 [1.52 - 1.59]	1.35 [1.32 - 1.38]
	Mixed	85.3 [84.9 - 85.7]	2.69 [2.60 - 2.79]	2.67 [2.58 - 2.77]	2.21 [2.14 - 2.30]
	Other	82.9 [82.6 - 83.2]	3.22 [3.15 - 3.29]	3.12 [3.05 - 3.18]	2.44 [2.39 - 2.50]
	Pakistani	79.6 [79.2 - 80.0]	3.99 [3.89 - 4.09]	4.12 [4.02 - 4.22]	3.59 [3.50 - 3.68]
	White other	87.7 [87.5 - 87.8]	2.20 [2.17 - 2.23]	2.24 [2.21 - 2.27]	1.93 [1.90 - 1.95]
IMD Quintile	1 (most deprived)	90.6 [90.6 - 90.7]	1.77 [1.76 - 1.79]	1.76 [1.75 - 1.78]	1.60 [1.59 - 1.62]
	2	92.1 [92.0 - 92.1]	1.48 [1.46 - 1.49]	1.47 [1.46 - 1.48]	1.34 [1.33 - 1.35]
	3	93.4 [93.4 - 93.5]	1.20 [1.19 - 1.22]	1.20 [1.19 - 1.21]	1.17 [1.16 - 1.18]
	4	94.0 [94.0 - 94.0]	1.09 [1.08 - 1.10]	1.09 [1.08 - 1.10]	1.09 [1.08 - 1.10]
	5 (least deprived)	94.5 [94.4 - 94.5]	1 (Ref)	1 (Ref)	1 (Ref)
Religion	Christian	93.8 [93.8 - 93.9]	1 (Ref)	1 (Ref)	1 (Ref)
	Buddhist	84.1 [83.5 - 84.7]	2.88 [2.76 - 3.01]	2.63 [2.52 - 2.74]	2.03 [1.95 - 2.12]
	Hindu	91.5 [91.2 - 91.7]	1.43 [1.39 - 1.47]	1.39 [1.35 - 1.43]	1.03 [1.00 - 1.06]
	Jewish	93.1 [92.8 - 93.3]	1.13 [1.09 - 1.18]	1.12 [1.08 - 1.17]	0.94 [0.90 - 0.97]
	Muslim	79.1 [78.9 - 79.4]	4.02 [3.95 - 4.09]	4.04 [3.97 - 4.11]	2.74 [2.69 - 2.79]
	No religion	91.9 [91.9 - 92.0]	1.34 [1.33 - 1.35]	1.25 [1.23 - 1.26]	1.23 [1.22 - 1.24]
	Other Religion	85.4 [84.9 - 85.8]	2.61 [2.51 - 2.71]	2.41 [2.32 - 2.50]	2.15 [2.07 - 2.23]
	Religion Not Stated	91.5 [91.4 - 91.6]	1.42 [1.41 - 1.44]	1.40 [1.38 - 1.41]	1.35 [1.33 - 1.36]
	Sikh	91.6 [91.3 - 91.9]	1.39 [1.34 - 1.45]	1.35 [1.30 - 1.41]	1.07 [1.03 - 1.11]
Household tenure	Owned	94.0 [93.9 - 94.0]	1 (Ref)	1 (Ref)	1 (Ref)
	Other	91.1 [90.9 - 91.2]	1.52 [1.49 - 1.55]	1.55 [1.52 - 1.58]	1.49 [1.46 - 1.52]

	Private rented	88.4 [88.2 - 88.5]	2.05 [2.02 - 2.07]	1.96 [1.94 - 1.99]	1.81 [1.79 - 1.83]
	Social rented	89.9 [89.8 - 89.9]	1.75 [1.73 - 1.76]	1.77 [1.76 - 1.79]	1.60 [1.59 - 1.61]
Rural-Urban	Rural	94.5 [94.4 - 94.5]	1 (Ref)	1 (Ref)	1 (Ref)
	Urban	92.8 [92.8 - 92.8]	1.33 [1.32 - 1.34]	1.34 [1.33 - 1.35]	1.12 [1.12 - 1.13]
Household composition	2 Elderly	94.5 [94.4 - 94.5]	1 (Ref)	1 (Ref)	1 (Ref)
	1 Elderly	92.5 [92.5 - 92.6]	1.38 [1.37 - 1.39]	1.53 [1.52 - 1.54]	1.32 [1.31 - 1.33]
	Care home	94.9 [94.8 - 95.0]	0.92 [0.89 - 0.95]	1.10 [1.06 - 1.13]	0.89 [0.86 - 0.91]
	Multigenerational	90.3 [90.3 - 90.4]	1.83 [1.82 - 1.85]	1.77 [1.76 - 1.79]	1.39 [1.38 - 1.40]
	Other (3+ Adults)	90.0 [89.6 - 90.3]	1.91 [1.84 - 1.97]	1.81 [1.75 - 1.88]	1.54 [1.49 - 1.60]

Note: Adults aged 70 years or over, living in England, alive on 15th March 2021. Model 1: unadjusted; Model 2: adjusted for sex and age (Cubic splines); Model 3: adjusted for sex, age (Cubic splines), care home status, rural/urban, region, ethnicity (except when looking at religion as an exposure), IMD quintile (except when looking at household tenure as an exposure), disability, BMI and comorbidities. See Table 1 for more details on the variables included in the models.

For peer review only

STROBE Statement—Checklist of items that should be included in reports of ***cohort studies***

	Item No	Recommendation	Page No
**Title and abstract**	1	(*a*) Indicate the study's design with a commonly used term in the title or the abstract	1
		(*b*) Provide in the abstract an informative and balanced summary of what was done and what was found	2
**Introduction**			
Background/rationale	2	Explain the scientific background and rationale for the investigation being reported	3
Objectives	3	State specific objectives, including any prespecified hypotheses	3
**Methods**			
Study design	4	Present key elements of study design early in the paper	3
Setting	5	Describe the setting, locations, and relevant dates, including periods of recruitment, exposure, follow-up, and data collection	3
Participants	6	(*a*) Give the eligibility criteria, and the sources and methods of selection of participants. Describe methods of follow-up	3
		(*b*) For matched studies, give matching criteria and number of exposed and unexposed	
Variables	7	Clearly define all outcomes, exposures, predictors, potential confounders, and effect modifiers. Give diagnostic criteria, if applicable	3
Data sources/ measurement	8*	For each variable of interest, give sources of data and details of methods of assessment (measurement). Describe comparability of assessment methods if there is more than one group	3
Bias	9	Describe any efforts to address potential sources of bias	3
Study size	10	Explain how the study size was arrived at	3
Quantitative variables	11	Explain how quantitative variables were handled in the analyses. If applicable, describe which groupings were chosen and why	4 Table S1
Statistical methods	12	(*a*) Describe all statistical methods, including those used to control for confounding	4
		(*b*) Describe any methods used to examine subgroups and interactions	NA
		(*c*) Explain how missing data were addressed	NA
		(*d*) If applicable, explain how loss to follow-up was addressed	NA
		(*e*) Describe any sensitivity analyses	NA
**Results**			
Participants	13*	(a) Report numbers of individuals at each stage of study—eg numbers potentially eligible, examined for eligibility, confirmed eligible, included in the study, completing follow-up, and analysed	3,4
		(b) Give reasons for non-participation at each stage	3
		(c) Consider use of a flow diagram	
Descriptive data	14*	(a) Give characteristics of study participants (eg demographic, clinical, social) and information on exposures and potential confounders	4 (Table1)
		(b) Indicate number of participants with missing data for each variable of interest	4 (Table 1)
		(c) Summarise follow-up time (eg, average and total amount)	
Outcome data	15*	Report numbers of outcome events or summary measures over time	Table 1

For peer review only - http://bmjopen.bmj.com/site/about/guidelines.xhtml

Main results	16	(*a*) Give unadjusted estimates and, if applicable, confounder-adjusted estimates and their precision (eg, 95% confidence interval). Make clear which confounders were adjusted for and why they were included<br>(*b*) Report category boundaries when continuous variables were categorized<br>(*c*) If relevant, consider translating estimates of relative risk into absolute risk for a meaningful time period	4
Other analyses	17	Report other analyses done—eg analyses of subgroups and interactions, and sensitivity analyses	
**Discussion**			
Key results	18	Summarise key results with reference to study objectives	5
Limitations	19	Discuss limitations of the study, taking into account sources of potential bias or imprecision. Discuss both direction and magnitude of any potential bias	5
Interpretation	20	Give a cautious overall interpretation of results considering objectives, limitations, multiplicity of analyses, results from similar studies, and other relevant evidence	5
Generalisability	21	Discuss the generalisability (external validity) of the study results	5
**Other information**			
Funding	22	Give the source of funding and the role of the funders for the present study and, if applicable, for the original study on which the present article is based	7

*Give information separately for exposed and unexposed groups.

Note: An Explanation and Elaboration article discusses each checklist item and gives methodological background and published examples of transparent reporting. The STROBE checklist is best used in conjunction with this article (freely available on the Web sites of PLoS Medicine at http://www.plosmedicine.org/, Annals of Internal Medicine at http://www.annals.org/, and Epidemiology at http://www.epidem.com/). Information on the STROBE Initiative is available at http://www.strobe-statement.org.

For peer review only
